# Many Functions of Telomerase Components: Certainties, Doubts, and Inconsistencies

**DOI:** 10.3390/ijms232315189

**Published:** 2022-12-02

**Authors:** Ion Udroiu, Jessica Marinaccio, Antonella Sgura

**Affiliations:** Dipartimento di Scienze, Università “Roma Tre”, Viale G. Marconi 446, 00146 Rome, Italy

**Keywords:** telomerase, gene regulation, mitochondria, apoptosis, glycolysis, stemness

## Abstract

A growing number of studies have evidenced non-telomeric functions of “telomerase”. Almost all of them, however, investigated the non-canonical effects of the catalytic subunit TERT, and not the telomerase ribonucleoprotein holoenzyme. These functions mainly comprise signal transduction, gene regulation and the increase of anti-oxidative systems. Although less studied, TERC (the RNA component of telomerase) has also been shown to be involved in gene regulation, as well as other functions. All this has led to the publication of many reviews on the subject, which, however, are often disseminating personal interpretations of experimental studies of other researchers as original proofs. Indeed, while some functions such as gene regulation seem ascertained, especially because mechanistic findings have been provided, other ones remain dubious and/or are contradicted by other direct or indirect evidence (e.g., telomerase activity at double-strand break site, RNA polymerase activity of TERT, translation of TERC, mitochondrion-processed TERC). In a critical study of the primary evidence so far obtained, we show those functions for which there is consensus, those showing contradictory results and those needing confirmation. The resulting picture, together with some usually neglected aspects, seems to indicate a link between TERT and TERC functions and cellular stemness and gives possible directions for future research.

## 1. Introduction

Since its discovery by Greider and Blackburn [1] (preceded by the prediction of its existence by Olovnikov [2]), telomerase has attracted the attention of many researchers, probably because of its unique, distinctive features: it is the only known eukaryotic-specific enzyme with reverse transcriptase activity, and it counteracts replicative senescence, potentially allowing cells to be immortal. Moreover, the idea that “normal” cells do not express telomerase and tumor cells do prompted the idea that a specific target to kill only cancer cells (without harming the normal ones) had been found [3]. The real picture, however, is far more complex, since many somatic cells express telomerase: not only hematopoietic stem and progenitor cells [4], but also many types of epithelial cells, such as keratinocytes [5] and lens epithelial cells [6].

In the last 20 years, however, the interest has also grown for the non-telomeric effects of “telomerase”, mainly focused on anti-apoptotic and anti-oxidant effects [7]. This is mirrored by the fact that dozens of reviews have been published (and many yet to come) on this issue. In reality, however, almost all of them are on the non-telomeric effects of TERT, the catalytic subunit of the telomerase complex. Very few studies (in proportion) looked for non-telomeric effects of the telomerase complex. This misunderstanding is not trivial, since it brings some authors (as we will show) to draw wrong conclusions from their experiments.

Although less studied than TERT, TERC (the RNA component of telomerase) has also been the subject of studies on non-telomeric functions, and (like its catalytic partner) is the protagonist of many reviews.

Gould [8] warned about the risks of relying on reviews and papers that cite experimental works without reading the original sources. Liang et al. [9] made a scientific survey indicating that this practice is present. This means that personal interpretations of the experimental studies of other researchers (which are legitimate) are disseminated as original, experimental proofs. In the field of the study of telomerase, this has happened many times and in some cases, as we will show, has led to the widespread belief in unrealistic phenomena.

The aim of this paper is not to make the umpteenth review repeating things already written many times, but to make a critical study of the evidence so far obtained on the non-telomeric functions of TERT and TERC. In order to do this, we have read and studied the original articles. In this way, we will show those functions for which there is consensus among experimental studies, those showing contradictory results and those needing confirmation. The resulting picture, together with some aspects that are usually neglected, will help to elucidate the state of the art non-telomeric functions of the telomerase components.

## 2. Telomerase: Function and Components

### 2.1. Telomerase Function

Telomerase is a reverse transcriptase that adds telomeric repeats at the 3′ ends of linear chromosomes [1]. These sequences are TTAGGG in most eukaryotes (and most probably represent the ancestral motif) but show many variations in several lineages [10]. Telomerase activity, through telomere elongation, helps to maintain genome stability, preventing chromosome ends from being recognized and processed as DNA double strand breaks [11].

Telomerase consists of a catalytic protein subunit with reverse transcriptase activity (TERT), and an essential RNA component known as telomerase RNA component (TERC) that contains a template for the synthesis of telomeric DNA [12]. The telomerase holoenzyme also contains additional proteins (dyskerin, NHP2, NOP10 and GAR1 in vertebrates) that play crucial roles in its biogenesis, localization, and regulation [13,14].

**Figure 1:**
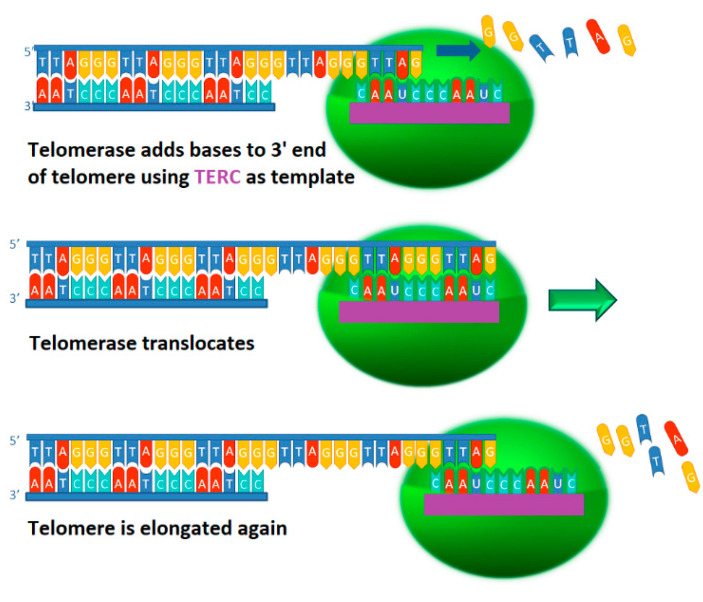
Telomere elongation by telomerase. After binding to the 3′ end of telomere, telomerase starts a cycle of elongation, translocation, elongation until the final dissociation (Image modified from Fatma Uzbas. “Working principal of telomerase” Licensed under: CC BY-SA 3.0).

The telomerase catalytic cycle comprises two main phases: the synthesis of a single telomere repeat, and the repositioning of the template for the synthesis of additional repeats (Figure 1). More precisely, it includes four stages: primer binding, elongation, translocation and dissociation [11].

The reaction starts by binding the DNA primer at the 3′ end of the telomere with the 5′ region of the RNA template of telomerase, which results in the formation of a short hybrid DNA/RNA duplex and then proceeds with the synthesis of one telomeric repeat. Once the repeat is completed, nucleotide addition arrests, followed by translocation of the RNA template. The translocation is a complex multi-step process and it has been shown to occur outside the active site [15]. Through this process, a single primer can be extended with numerous telomere repeats before complete disassociation from the telomerase enzyme [16,17].

For telomerase activity, TERT is usually considered the limiting factor, because in humans it is repressed in most somatic tissues, while TERC is constitutively expressed [18]. Although there is unanimous agreement on this concept, we think that it needs some clarification. It is true that tissues with no TERT expression show no telomerase activity (and not only in humans). However, while databanks for the expression of TERT in different types of cells and tissues are abundant, the same cannot be said for TERC (as for many other ncRNAs). Thus, so far, it is difficult to have a clear picture of cell- and tissue-specific TERC expression. Moreover, Avillon et al. [19] stated that TERC expression is a bad predictor of telomerase activity (while TERT expression is a good one), but they also found that TERC is expressed at very low levels in human normal brain, muscle and lung tissues and at low levels in breast and liver. More recently, Castle et al. [20] measured the expression of TERC in different human tissues, and we would like to highlight that the highest values are found in testes, just like TERT (Figure S1 in Supplementary Material). Moreover, Hartmann et al. [21] measured TERT and TERC expression in different tissues of the turquoise killifish (*Nothobranchius furzeri*), finding the same trend for both and their agreement with telomerase activity. Finally, it was also shown that TERC upregulation is an essential characteristic for telomerase induction in induced pluripotent cells [22]. Therefore, it can be summed up that TERC is expressed in more human tissues than TERT and in higher abundance, but also TERC expression shows significant differences between tissues and cell types.

### 2.2. TERT Structure

**Figure 2:**
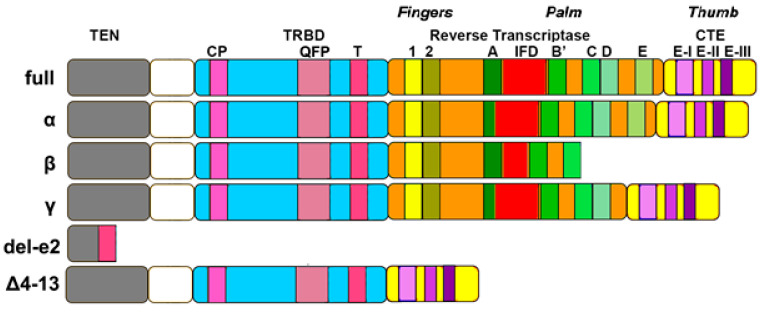
Architecture of of human TERT and alternative isoforms. TEN: telomerase essential N-terminal domain; TRBD: telomerase RNA binding domain; CTE: C-terminal extension. Isoform α shows a partial loss of Reverse Transcriptase-motif A. Isoform β shows partial loss and sequence variation of the IFD domain, as well as loss of the D and E fingers motifs and CTE domain. Isoform γ is similar to α, but also shows the loss of the E finger motif. Del-e2 is composed only of part of the TEN domain and T-motif.

The TERT protein is the catalytic component of the core of telomerase enzymes. This protein is composed of four conserved structural domains (Figure 2): the telomerase essential N-terminal domain (TEN), the telomerase RNA binding domain (TRBD), a central catalytic RT domain, and C-terminal extension (CTE).

The TEN domain is necessary for telomerase functioning in vitro and in vivo. The N-terminal contains several conserved telomerase-specific motifs, which are important for TERT–TERC binding interactions and for the rate of template copying during telomere synthesis [23,24,25]. This domain also contains ‘anchor’ sites that bind single-stranded telomeric DNA [26], allowing the complete delay disassociation of the DNA product from the enzyme and increasing repeat addition processivity. The TEN domains include RNA interacting domain 1 (RID 1), with a low-affinity binding site for the RT template/pseudoknot domain [27].

TRBD domain is unique to TERT protein, conferring the ability to use internal RNA templates upon addition of telomeric repeats. In addition, it contains RNA interacting domain 2 (RID 2) which has a high affinity for the three-way junction domain of TERC [28]. The protein-RNA interactions through RID1 and RID2 are essential for telomerase assembly [29]. The TRBD domain contains mainly helical motifs, including the CP, QFP and T motifs that participate in RNA binding [30,31].

The catalytic domain of TERT is the most characterized region of the protein and contains seven evolutionary conserved RT domains, essential for its enzymatic activities [23,32]. This domain has seven motifs: 1, 2, and A, B, C, D and E. The RT domain is described as a right hand divided in two subdomain that resemble the ‘fingers’ and ‘palm’ [33]. The finger domain helps to bind incoming nucleotides, while the palm represents the catalytic site [34]. Within the RT domain, a telomeric-specific motif (motif 3) is specific to high repeat addition processivity [35].

The CTE domain represents the so-called “thumb”, constituted by a helical bundle containing several surface-exposed loops that contributes to the formation and stabilization of an RNA-DNA heteroduplex in the enzyme active site [36]. Moreover, a nuclear export signal (NES) motif is present in these domains that allows TERT to exit through nuclear pores [37,38].

### 2.3. TERC Structure

A unique feature of telomerase is that the RNA template for DNA synthesis is an integral component of the holoenzyme. Being an ncRNA, TERC shows very little sequence conservation between species. In fact, mutations in ncRNA genes are not constrained by the genetic code and what is conserved is the secondary structure and not the sequence. Therefore, TERC is extremely difficult to find, even in not-so-distant species. So far, it has been found in ciliates [39], *Plasmodium* [40], land plants [41], Ascomycota [42], and metazoans [43].

**Figure 3:**
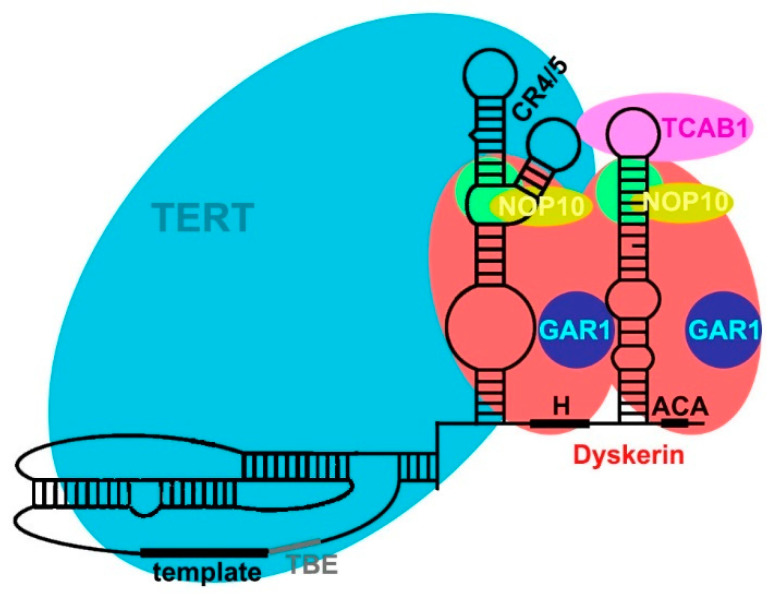
Human telomerase holoenzyme. TERC (structure in black) comprises the pseudoknot/template core domain (including template and template boundary element, TBE) and the CR4/5 domain (three-way junction), which are associated with TERT. In the scaRNA domain, two dyskerin molecules are bound to TERC through the H and ACA boxes. NHP2 (in green) and TCAB1 are also bound to TERC, while NOP10 and GAR1 are bound to dyskerin.

Despite no sequence conservation, TERC shows two domains conserved in all species: a pseudoknot/template core domain and a three-way junction domain [44]. Ciliate telomerase RNA, the simplest and shortest one, comprises only those. In yeasts (with the longest sequences), several other conserved domains are present, one of them binding to Ku and another to Sm proteins [42]. In echinoderms and vertebrates (Figure 3), the third one is the box H/ACA (typical of small Cajal body-specific RNAs, scaRNAs) domain [45].

**Figure 4:**
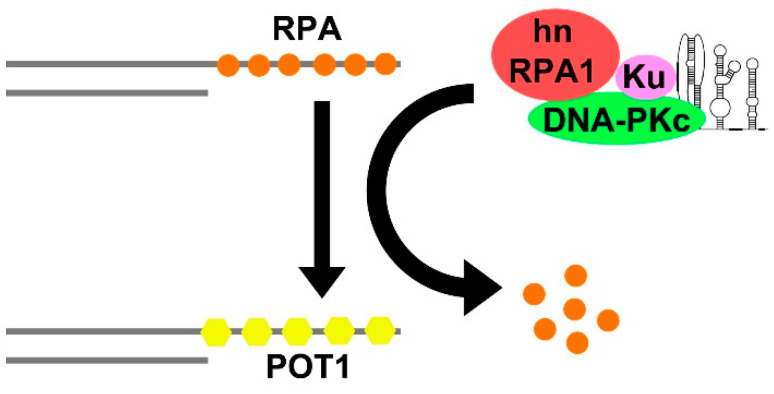
TERC collaborates in telomeric RPA to POT1 switch. TERC and DNA-PK (Ku + DNA-PKc) phosphorylate hnRNPA1; the latter removes RPA from telomeres, causing its substitution with POT1 and allowing telomere capping.

The core domain is essential for telomerase activity in vitro and in vivo [46]. This region contains the template for telomere addition, the 5′ boundary element, TERT binding site and a conserved pseudoknot structure [45,47]. The template sequences can be divided into a 5′ region encoding for telomeric DNA repeats and a 3′ region annealing to the DNA primer after template translocation. In addition, the template is near, on the 5′ end, to the template boundary element (TBE) that defines the end of the templating sequence (Figure 4). The pseudoknot contains a triple helix and the loss or disruption of this structure drastically reduces telomerase activity [48,49]. It is thought that its conformation is involved in the translocation of the template [15], which happens while the binding between TERT and the telomere is maintained. In summary, the TERC template/pseudoknot domain has a complex structure, necessary for the correct functioning of the holoenzyme [47].

A domain shared by all studied TERCs is a three-way junction distal to the template/pseudoknot domain, which is indispensable for enzymatic activity [50,51]. In animals [43], it is composed by the CR4/5 domain (Figure 3). With its L-shaped three-way-junction conformation, its two arms clamp onto the TRBD domain of TERT [52].

In animals [43], TERC has a conserved H/ACA domain located at the 3′ end (Figure 4). The H/ACA domain contains two stem-loops separated by a conserved H box that is located at the hinge region. The ACA box is located at the 3′-end and serves as binding site for dyskerin [53,54,55]. The bulge of the 3′ stem-loop of the H/ACA domain shows a Cajal body localization (CAB box) moiety, allowing the binding of TCAB1 protein [13,53,56]. This domain is essential for TERC stability, processing, nuclear localization and telomerase activity in vivo [46,57,58].

### 2.4. Secondary and Accessory Proteins

As we have seen, TERT binds only to TERC and to telomeres (including both telomeric DNA and telomeric proteins). TERC, instead, binds to different accessory proteins, essential to its localization, maturation, 3′ processing and ribonucleoprotein biogenesis [59]. TERC, through two stems present in the H/ACA domains (Figure 3), interacts with the protein complex formed by dyskerin, NOP10, NHP2, and GAR1 proteins [60].

Dyskerin is a nucleolar protein, fundamental for many cellular processes. The main function of dyskerin is to bind scaRNAs, thanks to the H and ACA boxes [61]. Dyskerin catalyzes the pseudouridylation of specific residues in ribosomal RNA and in small nuclear RNA [62,63,64,65]. In association with NHP2 and NOP10 (Figure 4), it forms a core trimer that directly binds to the H/ACA domain of TERC and regulates its stability [66]. GAR1, instead, binds only to dyskerin (Figure 3).

GAR1 is characterized by glycine–arginine-rich domains and is required for pre-rRNA processing [67]. NOP10 is characterized by a zinc ribbon domain in the N-terminal region [56]. Both NOP10 and GAR1 bind to dyskerin and are not directly bound to TERC [56,68]. Conversely, NHP2 is an RNA binding protein, but the specificity for the binding of H/ACA scaRNAs by NHP2 comes through its association with dyskerin via the small intermediate protein NOP10 [65].

Telomerase Cajal body protein 1 (TCAB1 or WRAP53) binds TERC for localization in the Cajal bodies, where the telomerase biogenesis to generate an active telomerase complex occurs [13,69].

Other proteins interact, temporarily, with telomerase; Hsp90, p23, pontin, reptin and others are required for the assembly of the telomerase complex and its translocation from the cytosol to the nucleus [70].

### 2.5. Has Telomerase Any Non-Telomeric Function?

#### 2.5.1. Addition of Telomeric Repeats at Double-Strand Break Sites?

As said before, dozens of reviews have discussed the extra-telomeric functions of “telomerase”. In reality, all these articles are about TERT, and not about the telomerase holoenzyme. The only known putative extra-telomeric function of this enzyme is its activity (addition of telomeric repeats) at double strand break (DSB) sites [71]. This is often cited in studies on karyotype evolution to justify the presence of interstitial telomeric sequences (ITS) [72]. The article by Flint et al. [71], however, did not investigate telomerase presence or activity at DSB sites, but studied telomeric sequences present on truncation breakpoints on the 16p chromosome arm of six patients. Their conclusion is based on the fact that, in five patients, the three or four nucleotides preceding the breakpoint are complementary to the TERC template. However, how can telomerase recognize a broken chromosome? Indeed, telomerase is recruited to telomeres by TPP1 [73], which, so far, is known to be present only in the shelterin complex.

In our view, the reason for the presence of telomeric repeats at “truncated” chromosomes is that, in reality, these are not truncated, i.e., the result of terminal deletions, but represent interstitial deletions. This hypothesis was already made by Furuya et al. [74] in order to explain the presence of telomeric sequences on the deleted short arms of chromosomes 9 and 10 in promyelocytic cell line HL-60. Moreover, Meltzer et al. [75] demonstrated that apparent terminal deletions characteristic of tumor cells and syndromes such as Miller–Dieker and Wolf–Hirschhorn are, in reality, subtelomeric translocations which were undetectable using conventional cytogenetics. These phenomena could be experimentally reproduced by X-irradiating fibroblasts (which do not express telomerase) and investigating whether or not some “truncated” chromosomes in reality show telomeric sequences (by Fluorescence In Situ Hybridization with a telomeric probe).

Nonetheless, telomeric sequences addition at broken chromosomes has been reported in a variety of species. McClintock [76] observed that dicentric-derived broken chromosomes acquire telomeres in maize. Fan and Yao [77] observed telomere formation during programmed chromosome breakage in *Tetrahymena thermophila*, and this was not dependent on the presence of a telomeric sequence at the break site. Matsumoto et al. [78] found novel telomeres on minichromosomes obtained by gamma-ray cleavage in the fission yeast *Schizosaccharomyces pombe*; they excluded the possibility of a recombinational event because “their chromosomal counterparts showed no sign of gross rearrangement”. Moreover, in the budding yeast *Saccharomyces cerevisiae*, Kramer and Haber [79] observed the de novo addition of telomeric sequence, but only when a telomeric sequence was present proximal to the break site, suggesting that this was needed as a primer for telomerase. Since they used an RAD52-deficeint strain, they concluded that recombination was impossible. However, in this same species, Wang and Zakian [80] observed telomere acquisition through a “novel recombination process involving a gene conversion event that requires little homology, occurs at or near the boundary of telomeric and nontelomeric DNA”; they suggested that this non-reciprocal recombination can also happen at non-telomeric ends, either through bypass or digestion until a telomere-like sequence. This work was seminal for the discovery of a new telomere maintenance mechanism called ALT (Alternative Lengthening of Telomeres). It should be added that Break-Induced Replication (BIR), one mode of action of ALT, can be both RAD52-dependent and -independent [81]. Therefore, in some of the species listed above, de novo telomere addition could be due to ALT and not telomerase activity. In any case, all these species being so phylogenetically far from vertebrates, whether or not telomerase activity at DSB is really possible in human cells should be investigated, as DSB, lacking TPP1, seems unable to recruit telomerase.

It should be noted, however, that the article by Flint et al. [71] was not about ITS, but about de novo telomeric sequences at truncated chromosomes. On an evolutionary timescale, these could fuse with other telomeric ends and give place to ITS, but this can also happen with normal telomeres without the need to find new mechanisms, involving telomerase or not.

#### 2.5.2. NOP2-Dependent Recruitment of Telomerase to Cyclin D1 Promoter

Recently, Hong et al. [82] identified NOP2 as a new TERC-binding protein found in catalytically active telomerase. They found that telomerase is recruited to the cyclin D1 (*CCND1*) promoter in a TERC-dependent manner through the interaction with NOP2, enhancing transcription of this gene, whereas TERT alone (i.e., in a TERC- and NOP2-independent manner) binds to the promoter of *Myc* [82]. The authors also provided a hypothesis, according to which the assembled telomerase complex can comprise either TCAB1 or NOP2. In the first case, telomerase is recruited to telomeres for their elongation; in the second one, it binds to the *CCND1* promoter. In any case, this mechanism of cyclin D1 activation is different from all the other cases of gene regulation, which are exerted by TERT without any other telomerase component (see next section, Section 3.1), and is the only case (so far revealed) of extra-telomeric function of telomerase.

## 3. TERT Non-Telomeric Functions

### 3.1. Gene Regulation

Several authors found that TERT can act as a transcriptional regulator modulating the expression of genes in different pathways. These are involved in most physiological processes, including cell cycle, metabolism, differentiation, cell signaling and cell survival. Overall, the transcriptional abilities of TERT seem ascertained, not only because authors from different research groups observed these features, but also because mechanisms through which this action is exerted have been elucidated. Indeed, it has been shown that some genes are activated through the direct interaction between TERT and their promoters, for example, RB/E2F. Alternatively, other genes are triggered in an indirect manner, for example NF-kB and Wnt, in which TERT binds different proteins involved in their signaling cascade [83,84].

#### 3.1.1. NF-κB Pathway

The transcription factor NF-κB (nuclear factor kappa B) regulates the expression of multiple genes involved in inflammation, immune response, cell proliferation, differentiation and apoptosis. It is also known that the transcription factor NF-κB binds 350 bp upstream of TERT and activates its transcription [85,86]. On the other hand, it has been demonstrated that TERT stimulates the expression of several genes whose transcription is controlled by the NF-κB pathway, and TERT interacts directly with the NF-κB p65 subunit, regulating its transcription and leading to the recruitment of a subset of NF-κB promoters such as Interleukin 6 (IL6) and TNF alpha [84,87]. These cytokines, fundamental for inflammation and cancer progression, together with NF-κB, can, in turn, transcriptionally upregulate telomerase levels [87]. These findings support a functional interplay between TERT and NF-κB, further reinforced by the observation of an enhanced expression of NF-κB target genes. Thus, it is interesting to observe that NF-κB serves as a transcriptional inducer of TERT, and vice versa, highlighting the positive feedback regulation between NF-κB and TERT. This regulatory loop may be one of the mechanisms underlying the telomerase activity typical of human cancers.

#### 3.1.2. Wnt/β-Catenin Pathway

Besides NF-κB, TERT has also been reported to regulate the transcriptional activity of the Wnt/β-catenin complex. The Wnt/β-catenin signaling pathway is a central regulator of embryogenesis and the self-renewal property of adult stem cells in proliferating tissues, such as cell proliferation, cell polarity and cell fate determination, but it is also involved in the variety of development disorders and cancers [88]. The first connection between TERT and Wnt/ β-catenin pathway was discovered by Artandi’s group [89]. They described the way that TERT interacts with BRG1 (now known as SMARCA4), a chromatin remodeling protein, and activates Wnt-dependent genes. The authors showed that TERT physically occupies Wnt/β-catenin dependent promoters including those of cyclin D1 and *Myc* [89]. On the other hand, Liu and colleagues [90] did not see a physical association between TERT and SMARCA4. Instead, they found that TERT is able to cause a TGF-B1-mediated β-catenin induction and its nuclear accumulation, thus interacting with β-catenin in the transcriptional regulation of downstream targets [90].

#### 3.1.3. pRb and Cyclins

D-type cyclins (D1, D2 and D3), which are regulators of G1 phase progression and are part of the pRB/E2F pathway [91], have been showed to be regulated by TERT. Different authors, employing different cell lines, showed that TERT increases cell growth and stimulates proliferation [92,93,94]. In particular, TERT has been shown to induce hyperphosphorylation of pRB, increasing E2F transcriptional activity, and thus increasing the number of cells in S phase [94].

#### 3.1.4. Ribosomal DNA

Gonzalez et al. [95] showed that TERT binds to ribosomal DNA (rDNA) and stimulates its transcription by RNA polymerase I (Pol I) during liver regeneration and Ras-induced hyperproliferation, but not under normal conditions (e.g., TERT-expressing fibroblasts). Moreover, they showed that TERC co-immunoprecipitates with Pol I, and concluded that the whole telomerase complex stimulates rDNA transcription. This was corroborated by the fact that TERT or TERC abrogation reduces Pol I transcription. However, while TERT-linked stimulation of Pol I transcription was seen in fibroblasts only in the presence of Ras, TERC co-immunoprecipitated with Pol I in (hTert-positive, non-Ras-transformed) HEK293 cells. Thus, is it possible that TERT binding to Pol I is Ras-dependent and TERC binding to Pol I is Ras-independent? Or, put in another manner, TERC is “normally” bound to rDNA/Pol I and in conditions of hyper-proliferation (e.g., expression of Ras) TERT also binds to them. Using fibroblasts (Ras- and non-Ras-expressing) to repeat the experiment carried out with HEK293 will answer this question.

#### 3.1.5. RNA Polymerase III Target Genes

Performing ChIP sequencing (ChIP-seq) to determine genome-wide TERT occupancy, Khattar et al. [96] found enrichment in several genomic regions, and 60% of them were regulated by RNA polymerase III (Pol III), i.e., tRNAs, 5S rDNA, 7SL RNA, SINE and LINE elements. Moreover, they found that endogenous TERT directly associates with the Pol III subunit RPC32 and enhances its recruitment to chromatin, and that ectopic expression of TERT results in increased transcription of tRNAs. Interestingly, in the figures of that article [96], we noted that *RMRP* (a ncRNA transcribed by Pol III), *ILF2* and *POLG* (transcribed by Pol II) are also bound by TERT: the presence of *POLG* (encoding the catalytic subunit of mitochondrial DNA polymerase) is interesting for the mitochondrial roles of TERT (see Section 3.3), while RMRP is linked to the controversial RNA polymerase activity of TERT (see Section 3.2).

#### 3.1.6. Transcriptomic Studies

Several studies have investigated gene expression following silencing or over-expression of TERT. However, in almost all of them, it is impossible to discriminate between the effects of telomerase activation/deactivation and those intrinsically due to TERT alone. Nonetheless, a few authors used TERC-deficient cells, in which telomerase-mediated effects can be excluded (Table S1 in Supplementary Material). Liu et al. [97], using TERC-lacking WI-38-VA13 (immortalized fibroblasts) and U2OS (osteosarcoma) cells, found TERT-induced upregulation of genes promoting cell adhesion and migration. Interestingly, we also noted *CDKN2B* (p15) among the upregulated genes and lncRNAs NEAT1 and MALAT1 among the downregulated genes. Among the upregulated genes found by Jaiswal et al. [98] after ectopic expression of TERT in U2OS, there are some Wnt- and TGF-b-related, but also *CDKN2B*. Among the downregulated ones, instead, several differentiation-linked genes (such as keratins) are present. Finally, using the same cells (but also HeLa), and performing a proteomic study, the same authors [99] found upregulation of Hsp60, Hsp70, Hsp90, and GAPDH.

### 3.2. An RNA-Dependent RNA Polymerase?

Being a reverse transcriptase, TERT can be described as an RNA-dependent DNA Polymerase. However, Maida et al. [100] proposed that TERT can also act as an RNA-dependent RNA polymerase (RdRP). Using fibroblasts, HeLa and MCF7 cells, they reported that human TERT forms a complex with RMRP (the RNA component of another ribonucleoprotein, mitochondrial RNA processing endoribonuclease): the latter is used as both the substrate and the template for RdRP activity via 3′ end loop back, resulting in a double-stranded RNA (dsRNA) formed by sense + antisense RMRP. This is then processed by the endoribonuclease dicer into small interfering RNA (siRNA), which controls RMRP endogenous levels [100]. Thus, they described a negative feedback, where siRNAs derived from ds RMRP inhibit the expression of RMRP. This was demonstrated by the fact that ectopic expression of TERT reduced the level of RMRP [100].

However, the Cech Lab was unable to reproduce the TERT-dependent RdRP activity for full-length duplex (E. Podell and T. Cech, personal communication). Moreover, Mattijssen et al. [101] used the siRNA sequence of Maida and colleagues and found no effects on RMRP levels in HeLa cells. As said above, Maida et al. [100] showed that overexpression of TERT reduces the expression of RMRP; however, available transcriptome data (although comprising too few samples to make statistical analyses, see Figure S2 in Supplementary Material) seem to show that IMR90 and WI-38-VA13 fibroblasts ectopically expressing TERT have higher (and not lower) levels of RMRP compared to their normal counterpart. This observation made by us would be in agreement with the abovementioned (see Section 3.1.5) binding of TERT on an RMRP promoter (and probably increased transcription) observed by Khattar et al. [96].

Therefore, the article by Maida et al. [100] should be confirmed by other independent laboratories. In any case, there are intriguing links between telomerase and RNase MRP (Figure S3 in Supplementary Material). In yeast, Pop1, Pop6 and Pop 7, which are protein subunits of RNase MRP, are also indispensable for telomerase proper assembly and activity in vivo [102]. Indeed, Tlc1 (yeast ortholog of TERC) shows a stem with a Pop6/Pop7 binding domain, which is similar to a corresponding domain in Nme1 (yeast ortholog of RMRP). In vertebrates, however, this stem is absent and, so far, TERT is not associated with any subunit of RNase MRP (Figure S3 in Supplementary Material). Nonetheless, we found some similarities between the Pop6/Pop7 binding domain of RMRP and the CR4/5 domain of TERC, but this would need further investigation.

Finally, Fujita et al. [103], using a combination of engineered DNA-binding molecule-mediated chromatin immunoprecipitation and RNA sequencing, also found RMRP among the ncRNAs associated with telomeres. They described RMRP as a “known telomere-binding ncRNA” and cited the article of Maida et al. [100]. The latter, in turn, never showed that the putative TERT-RMRP complex was present at telomeres. Therefore, the finding of Fujita et al. [103] would need further investigation to understand the presence of RMRP at telomeres, and its possible association with other proteins.

### 3.3. TERT and Mitochondria

#### 3.3.1. Shuttling of TERT between Nucleus, Cytoplasm and Mitochondria

Many authors have showed that TERT is also localized in the mitochondria [104,105]. The first evidence that TERT has a mitochondrial role came from the identification of a mitochondrial-targeting signal (MTS) at its N-terminus, composed by 20 amino acid residues [104]. The MTS sequence is sufficient and indispensable when targeting TERT to mitochondria; in fact, a mutation of two amino acid residues of the MTS prevented its mitochondrial localization. The authors found that the MTS is conserved among “higher” eukaryotes such as plants and mammals but not present in “lower” species such as yeast and *Tetrahymena* [104]. These data have been interpreted by Saretzki [106] as evidence that this function has been acquired rather late in the evolution of the enzyme. However, mammals are more phylogenetically related to yeasts than to plants, and we think that this issue needs further studies.

To understand the molecular function of mitochondrial TERT, it is necessary to define its submitochondrial localization, that is to say its presence in the mitochondrial membrane or in the mitochondrial matrix. Haendeler and co-workers [107], with different experimental systems, found that TERT protein is imported into mitochondria via the translocases of outer and inner membranes and resides mainly in the mitochondrial matrix. Indeed, the authors demonstrated that overexpressed myc-tagged TERT co-immunoprecipitated with TOM 20, TOM 40 and TIM 23 [107]. These proteins are translocases of the outer (TOM 20 and TOM 40) and inner (TIM 23) mitochondrial import machinery, suggesting an active mitochondrial import mechanism [108].

On the C-terminus, instead, TERT shows a nuclear export signal (NES) involved in cellular traffic. In fact, NES interacts with the nuclear export receptor exportin, and so it is actively transported through nuclear pores [37,38]. Seimiya et al. [37] demonstrated that 14-3-3 protein regulates the nuclear localization and accumulation of TERT, through the binding of the NES signal. In contrast, interrupting the interaction of both proteins and mutation of NES resulted in a cytoplasmic localization of TERT.

The intracellular distribution of TERT can be regulated through the activation of many proteins involved in its shuttling between nucleus, cytoplasm and mitochondria [109,110]. It is interesting to note that, under oxidative stress, the distribution of TERT is regulated by posttranslational modifications. Indeed, Src kinase phosphorylates TERT at tyrosine 707 (Y707) under oxidative stress, which allows TERT binding to Ran GTPase and consequent nuclear export via exportin [109]. On the other hand, Büchner et al. [110] have showed that levels of wild-type (wt) TERT decrease in the mitochondria upon H_2_O_2_ exposure, but no change occurs in the mutant of TERT (Y707F) that is not phosphorylated by Src kinase. Thus, they suggested that Src kinase negatively influences TERT mitochondrial import upon oxidative stress [110].

#### 3.3.2. TERT Association with mtDNA

Haendeler et al. [107] first showed that TERT interacts with mtDNA, in particular in two coding regions around the *ND1* and *ND2* genes of complex I, suggesting that this binding mechanism could be responsible for the protection of mtDNA from ethidium bromide-induced damage in murine fibroblasts and HEK293 cells. They further explained the presence of TERT near *ND1* and *ND2* by the fact that there are telomeric sequences near these genes [107]. However, there are 22 telomeric sequences distributed across the two strands of the mitochondrial genome [104], so there is no reason why TERT should interact only with these two regions. These authors also observed that HEK293 cells overexpressing TERT had a better respiration capacity and more activity of the complex I, compared to cells with a catalytically inactive mutant TERT. They finally concluded that the catalytic activity of TERT is necessary for the improvement of the respiratory chain activity and may be responsible for protecting mtDNA [107].

Sharma et al. [111] confirmed the import of TERT into the mitochondrial matrix of HEK293 cells. In addition, they demonstrated the ability of TERT to interact with additional regions of mitochondrial DNA coding for ribosomal 12S and 16S RNAs, ND1, 2,4,5, and COX I and III, as well as various tRNAs and subunit 6 and 8 of ATP synthase [111]. These results suggested that TERT binds to mtDNA non-specifically (directly or indirectly, and independently from the presence of telomeric sequences), or that it binds to mtDNA via a widely distributed cis-element.

However, TERT binds to telomeric sequences only if it is recruited by the telomeric protein TPP1 [73], which has been shown to be absent in mitochondria [112]. Moreover, we have seen that TERT can regulate expression of nuclear genes by interacting with their promoters (Section 3.1) and this has nothing to do with the presence or absence of telomeric sequences. Therefore, we think that there is no need to look for telomeric sequences in the mitochondrial DNA to explain the ability of TERT to regulate mitochondrial genes.

In their study on TERT and RMRP (Section 3.2), Maida et al. [100] also found that TERT is associated with mitochondrial tRNAs (mt-tRNAs). Sharma et al. [111], using purified mitochondria, confirmed the association of TERT with mt-tRNAs, as well as with RMRP. Moreover, they demonstrated in vitro that TERT is able to perform reverse transcription using tRNAs as primers. Since it has been proposed that priming of the light strand origin for mtDNA replication may rely on activity of a reverse transcriptase [113], they suggested that TERT may be involved in mtDNA replication [111]. On the other hand, Balasubramaniam et al. [114] showed that mt-tRNA genes can act as alternative origins of replication. They suggested that TERT may bind to the light strand origin of replication, inhibiting its activity, and increase the alternative use of mt-tRNA genes as origins of replication, thus reducing the contribution of the deletions-prone strand displacement mode to the replication of mtDNA.

#### 3.3.3. Controversial Effects of TERT in Mitochondria

So far, opposite results have been obtained on the role of TERT oxidative stress-mediated mtDNA damage [104,105,115].

The first study, performed by Santos et al. [104], showed that wt TERT increases H_2_O_2_-mediated mtDNA damage in NHF fibroblasts and HeLa cells. Subsequently, using a non-mitochondrial mutant (R3E/R6EhTERT) stable expressed in the cells, the same authors demonstrated the complete abolition of mtDNA damage caused by H_2_O_2_. The same results were obtained (in NHF and MRC-5 fibroblasts) using a catalytically inactive TERT mutant, prompting the authors to conclude that the induction of mtDNA damage relies on the reverse transcriptase activity of TERT [105]. These data led to the conclusion that wt TERT-expressing cells have more mtDNA damage, and that TERT must be catalytically active in mitochondria in order to promote H_2_O_2_-induced mtDNA damage [104,105].

However, Ahmed et al. [115] contradicted these findings, using MRC-5 fibroblasts. The authors proved that cells overexpressing TERT had less damage compared to the normal counterpart after H_2_O_2_ treatment. Similar findings were obtained with cells under hyperoxia (40% oxygen). In TERT overexpressing cells, the mtDNA damage was completely eliminated after 40 days (when TERT was localized exclusively in mitochondria), whereas in normal cells hyperoxia-induced mtDNA damage increased for 20 days and then plateaued [115]. Therefore, the authors found less mitochondrial DNA damage after both acute and chronic oxidative stress. On the other hand, a decrease in endogenous TERT levels in human umbilical endothelial cells (HUVEC), using anti-TERT siRNAs, significantly increased the levels of mitochondrial superoxide and intracellular peroxides [115]. Ahmed and co-workers have concluded that the cells overexpressing TERT showed an improved mitochondrial function and, specifically, less mitochondrial superoxide production and lower levels of cellular ROS.

### 3.4. Cellular Effects

So far, we have dealt with mechanistic studies investigating the non-telomeric functions of TERT. An equivalent (or greater) amount of research has been performed, instead, investigating the cellular effects of TERT. Indeed, most of the first studies on the non-telomeric functions of TERT showed that its downregulation induces apoptosis in cancer cells [116,117,118]. In particular, it has been shown that depletion of TERT induces the activation of pro-apoptotic protein Bax and caspases [118,119]. Subsequently, it was shown that TERT, although not preventing stress-induced senescence, protects normal human fibroblasts from apoptosis and necrosis [120]. Therefore, the anti-apoptotic properties of TERT are exerted both in cancerous and non-cancerous cells.

Different authors showed that TERT lowers the levels of mitochondrial ROS in several types of cells, both untreated and H_2_O_2_-treated [107,115,121].

Moreover, Kovalenko et al. [121] generated a transport deficient TERT mutant, defective in the nuclear export signal (NES, see Section 3.3.1), through changing amino acids 980 and 987. As expected by the authors, cells overexpressing _NES-_hTERT showed mitochondrial dysfunction, which was absent in wt TERT-transfected cells [121]. These data also agreed with the idea of the essential role of subcellular shuttling of TERT in protecting the mitochondria from oxidative stress [105,107,115,121].

The same defective _NES-_hTERT mutant has been utilized by Santos’ group, in two cancer lines; these showed decreased proliferation and increased levels of mtDNA damage compared to parental cell lines [122]. This finding further confirmed that a lack of TERT within mitochondria promotes damage to the mtDNA, while no mtDNA damage was observed when TERT was in the mitochondria.

Sharma et al. [111] used a TERT mutant with defective MTS (see Section 3.3.1), thus unable to enter mitochondria. While the fibroblast with wt TERT showed reduced mtDNA damage and mitochondrial superoxide generation, the opposite was observed in fibroblasts with mutant TERT [111].

Indran et al. [123] demonstrated that TERT decreases mitochondrial superoxide and cellular peroxide through an increase in the activity of the glutathione pathway enzymes.

Moreover, Indran et al. [123] observed that, after H_2_O_2_ treatment, TERT overexpressing cells blocked the Bax translocation as well as the release of other pro-apoptotic proteins, causing increased survival after oxidative stress [123].

In agreement with the abovementioned studies, Martens et al. [124] confirmed that the overexpression of TERT in MRC-5 fibroblast protects mtDNA from different oxidative stress damage. However, they found that this is not due to an increase in mtDNA repair, but to an increase in antioxidant defense mechanisms to prevent mtDNA damage. Specifically, Martens et al. [124] found that TERT induces an increase in the levels of manganese superoxide dismutase (MnSOD) and forkhead-box-protein O3 (FoxO3a) proteins, which are both encoded by nuclear genes.

Finally, Zhang et al. [125] found that TERT expression increases in cisplatin treated osteosarcoma cells, and that TERT translocates from the nucleus to mitochondria, inhibiting apoptosis and improving mitochondrial function via alleviating intracellular ROS. One of the three osteosarcoma cell lines was U2-OS, in which Zhang et al. [125] identified TERT mRNA through qRT-PCR, and TERT protein through Western Blot in mitochondrial fraction and through immunofluorescence in confocal images. However, U2-OS is well-known for being an ALT (Alternative Lengthening of Telomeres) cell line devoid of any TERT expression [97,98,126]. Therefore, the whole study seems dubious to us.

Taken together, almost (but not all) studies conducted so far show a wide consensus for the anti-apoptotic and antioxidant properties of TERT. Nonetheless, the mechanisms by which these properties are exerted need to be elucidated, as some studies showed a direct intervention of TERT into mitochondria, and others proposed a modulation of the antioxidant mechanisms, which are nuclear-encoded, and so do not involve the possible entry of TERT into these organelles.

### 3.5. Alternative Isoforms of TERT and Their Functions

Although the study of the mechanisms that regulate alternative splicing of TERT is still just beginning, it is clear that several isoforms of this protein exist [127]. This alternative splicing takes place mainly by exon-skipping, but also intron-retention, and is regulated by SRSF and hnRNP protein families [128]. To date, only full-length TERT retains reverse-transcriptase activity and no alternative splice variant exhibits catalytic activity [127].

Isoform α (resulting from a partial in-frame deletion of exon 6) shows a partial loss of Reverse Transcriptase (RT)-motif A (Figure 2). Isoform β shows a 182 bp out-of-frame deletion [129], resulting in a partial loss of the Insertion in Fingers Domain (IFD) and introducing a premature termination codon, determining truncation and loss of RT-motifs D and E, as well as loss of the CTE domain. Isoform γ is similar to α, but also shows the loss of the RT- motif E. An isoform with the deletion of exon 2 (del-e2) retains only the first part of TEN domain and the T-motif of TRBD [130]. Another isoform called Δ4–13 (loss of exons 4–13) shows loss of the RT domain, but perfect conservation of the other ones [127]. Finally, isoforms with partial retention of intron 14 (called INS3 and INS4) have been identified: they show premature stop codons leading to truncation [131].

Isoforms α, γ, INS3 and INS4 act as dominant negative inhibitors of telomerase activity [131,132,133]. Although the relatively low transcript abundance of these isoforms could mean that they are insufficient to inhibit telomerase activity in immortal cells, they could play such role in normal tissues. Indeed, their expression is highly tissue-specific and developmentally regulated [134].

Listerman et al. (2013) reported that isoform β can also inhibit telomerase activity, in this case by sequestering TERC. However, since TERC is expressed at much higher levels (two orders of magnitude) than TERT, it seems difficult to us that this phenomenon actually takes place. Moreover, the same authors showed that this isoform, as well as full length TERT, reduces cisplatin-induced apoptosis in cancer cells [128]. Finally, they also showed that isoform β associates with mitochondria (as assessed by immunofluorescence). Furthermore, this last finding is quite mysterious to us, since this isoform lacks the CTE domain (Figure 2) where the nuclear export signal (NES) is located (Seimiya et al., [37], see Section 2.2). Thus, if isoform β cannot exit from the nucleus, how could it go to the mitochondria? An explanation for this contradiction could be that TERT isoform β, after its translation in the cytoplasm, remains there and is not imported into the nucleus. This could explain how isoform β goes to the mitochondria [128], but not how it sequesters TERC [128], which resides in the nucleus. Therefore, there should be a nuclear pool (which cannot re-exit from the nucleus) and a cytosolic pool (which can be moved to the mitochondria) of isoform β.

Isoform Δ4–13 (which is present in both telomerase-positive and negative human cells) enhances cell proliferation and LiCl-induced Wnt signaling when overexpressed in telomerase-negative cell lines [127].

Zhdanov et al. [135,136] studied the effects of Endonuclease G (EndoG) on splice variants of TERT in CaCo2 cells and n CD4+ T cells. EndoG is normally located in the mitochondria (where it is involved in replication), but under oxidative stress it relocates to the nucleus, participating in the apoptotic process [137]. Zhdanov et al. [135,136] observed that the enhanced expression of EndoG increases the expression of isoform β at the expense of full length TERT. Interestingly, nuclear relocalization of EndoG is determined by its dissociation from Hsp70 and STUB1 [138], which, respectively, impedes nuclear import of TERT after translation and degrades TERT in the cytoplasm [139]. Moreover, cisplatin treatment was found to modulate NOVA1 [140], which, in turn, regulates TERT splicing and generating isoform β at the expense of full length TERT [141].

It is also interesting to see the expression levels of TERT isoforms measured by Zhdanov et al. [136] in CaCo2 cells. In untreated control cells, full length TERT is the predominant isoform in the nucleus and cytoplasm, while in mitochondria its abundance is equivalent to isoform β. Following cisplatin treatment (and consequent relocalization of EndoG from mitochondria to cytoplasm and nucleus), full length TERT decreases both in nucleus and cytoplasm, while isoform β increases. Interestingly, in mitochondria, full length TERT increases and isoform β decreases. In all cases, isoform α was nearly undetectable. Thus, EndoG-mediated apoptosis not only increases transcription of isoform β at the expense of full length TERT (as measured by mRNA), but also causes the entry of full length TERT into the mitochondria and the exit of isoform β from them.

Overall, these results confirm that TERT is present in mitochondria, as previously shown by other authors ([104,110,128]; see Section 3.3.1). Moreover, they show that entry/exit to/from mitochondria differs for the different TERT isoforms. This could be at the basis of the conflicting results about the pro- or anti-apoptotic role of mitochondrial TERT ([104,115]; see Section 3.3.3).

Although the study of alternative isoforms shows many technical difficulties, it seems a promising field for understanding the non-canonical functions of TERT. How many of these are indeed exerted by alternative isoforms? Most probably, the ontogenetically and tissue regulated switch from some isoforms to other ones could be linked to different non-canonical functions.

## 4. TERC Non-Telomeric Functions

### 4.1. Gene Regulation

Some authors have studied gene expression following the suppression or ectopic expression of TERC. Some studies were performed in cancer cells (with active telomerase), but there are also some that used primary cells or U2OS.

Following the silencing of *TERC* in cancer cells such as HCT116 [142], HeLa ([143], and murine melanoma [144], similar results have been observed: downregulation of genes involved in proliferation (cyclins, oncogenes, DNA repair proteins, chromatin regulators, transcription factors, ribosomal genes) and in the glycolytic pathway, and upregulation of genes involved in differentiation (Table S2 in Supplementary Material).

Another study is very interesting, in our view, since TERC was ectopically expressed in U2OS cells [145], which normally show neither TERC nor TERT, therefore eliminating any confounding factor. Altered expression of 431 genes, with high enrichment of those involved in cellular immunity, was found. Moreover, performing genome-wide screening using a previously identified ‘binding motif’ of TERC, Liu et al. [145] identified 14 genes that are transcriptionally enhanced by TERC, through its association with the promoter of these genes through forming RNA–DNA triplexes. Four of these genes (*LIN37*, *TPRG1L*, *TYROBP* and *USP16*) stimulate the activation of the NF-κB pathway. This has been suggested to be linked the immunodepression of several forms of dyskeratosis congenita caused by TERC defect cells [145].

Comparing bone marrow stromal cells with siTERC to their normal counterpart, Balakumaran et al. [146] observed a decrease in hematopoietic factors (recapitulating the bone marrow failure of patients with Dyskeratosis congenita). Their RNA-seq data also showed a significant upregulation of the p53/apoptosis pathways, as well as an increase in *ATR* (sensor of DNA single strand breaks), *TIGAR* (an inhibitor of glycolysis) and several histones of the types 1 and 2, and significant downregulation of chromatin regulators, transcription factors and DNA repair genes. Similar results were obtained by Gazzaniga and Blackburn [147], who silenced *TERC* in stimulated CD4 T cells, by Sung et al. [148] in *TERC*^-/-^ embryonic stem cells, and by Kedde et al. [149] using a panel of several cell lines.

Chu et al. [150] performed Chromatin Isolation by RNA Purification (ChIRP)-seq of TERC in HeLa S3 cells transduced with TERC, and identified over 2198 TERC binding sites in the genome. Since TERT can bind to and activate Wnt target genes [89], the authors hypothesized that TERC “*as a component of the TERT complex*” may also co-occupy some of the same genes. However, TERC is a component of the *telomerase* complex, and Wnt target genes are instead activated by the TERT-SMARCA4 complex (see Section 3.1). Nonetheless, the effective binding of TERC to predicted promoters could be a promising subject for future studies.

Finally, Ivanyi-Nagy et al. [151] mapped the RNA interactome of TERC and identified a set of non-coding and coding TERC-interacting RNAs, including the histone 1C mRNA (HIST1H1C). Disruption of the TERC-HIST1H1C RNA association resulted in markedly increased telomere elongation without affecting telomerase enzymatic activity. Although this study was not about gene regulation, it underlines another possible way in which TERC affects gene expression, i.e., interaction with mRNAs rather than the activation/repression of promoters.

Probably most of the effects listed above are indirect, and association between their promoters and TERC has only been shown for a few genes [145]. Nonetheless, apart from the expression of histones (which show very contradictory results in the previous studies), the results obtained from different authors give a coherent picture (Table S2 in Supplementary Material): TERC seems to promote proliferation (cyclins, cyclin-dependent kinases, mitotic initiators, DNA replication) and glycolysis, and to inhibit apoptosis and differentiation. It is noteworthy that sustained proliferation, glycolysis, and de-differentiation are markers of stemness, thus suggesting a specific role for TERC in stem and cancer cells.

### 4.2. New Actors in the Plot

#### 4.2.1. RPL22

An interesting case comes from Marek’s disease virus (MDV), an alphaherpesvirus that causes T cell lymphomas in chickens, and which harbours two copies of TERC [152]. These show 88% identity with chicken (*Gallus domesticus*) TERC and, on a phylogenetic tree, chicken TERC is more closely related to MDV than to any other bird, indicating, in our view, that MDV obtained these genes by horizontal transfer. MDV mutants lacking TERC are able to replicate normally, but do not induce lymphomas in their hosts [152]. Thus, MDV TERC is needed for tumorigenesis and not for viral replication. Kaufer et al. [153] observed that disruption of the interaction between MDV-TERC and chicken TERT (by mutating the p6.1 stem in the CR4/5 domain of TERC) delayed the onset of lymphomagenesis, but did not abrogate it. Therefore, the increase of telomerase activity driven by MDV-TERC accelerates disease progression, but is not essential for tumorigenesis, indicating telomerase-independent functions of MDV-TERC [153]. Substituting MDV-TERC with chicken TERC, Kheimar et al. [154] demonstrated the same pro-oncogenic activity, showing that this property is not restricted to MDV-TERC, but is shared by its vertebrate counterpart.

Investigating the telomerase-independent, pro-oncogenic properties of MDV-TERC, Kaufer et al. [155] discovered that RPL22 (60S ribosomal protein L22, involved in T-cell development and virus-induced transformation) directly interacts with MDV-TERC (both wild-type and TERT-incompetent mutant) and is relocalized to the nucleoplasm. It is noteworthy that *RPL22* is among the genes we previously observed to be upregulated by TERC (see Table S2 in Supplementary Material), and it was previously demonstrated that, in human cells, RPL22 is bound to TERC [156]. In order to demonstrate the role of RPL22, Kheimar and Kaufer [157] deleted TERC from MDV, thus abolishing tumorigenesis, and showed that its substitution with EBER-2 (Epstein-Barr virus-encoded RNA 2) restored tumour formation in MDV that lacked TERC. EBER-2 is known to bind to RPL22 (as well as to La and Pax-5) and to drive its relocalization to the nucleoplasm [158].

All this evidence shows a new function of TERC: an interaction with RPL22, and a protein with extra-ribosomal functions linked to splicing control, proliferation and differentiation.

#### 4.2.2. DNA-PK

An important protein involved in the repair of DNA double-strand breaks, through non-homologous end-joining, is the DNA-dependent protein kinase (DNA-PK). DNA-PK is composed of a DNA-binding subunit, the Ku heterodimer (composed of Ku70 and Ku80) and the catalytic subunit DNA-PKcs [159]. It is well established that, in yeasts, attachment of the Ku heterodimer to TLC1 (TERC homologue) is required for the proper recruitment of telomerase to the chromosome end and telomere synthesis [160]. This is not the case in vertebrates [45]. Nonetheless, Ting et al. [161] found that human Ku70/80 interacts directly with TERC, binding its 3′-terminal region (nt 404–451). While in yeasts the same site of Ku can bind double-strand DNA and RNA [162], in human Ku there are two different sites [163]. Thus, in yeasts, Ku cannot bind TLC1 and telomeres at the same time and recruitment of telomerase to telomere is proposed as a “hand-off” model, in which Ku recruits then passes telomerase to the shelterin proteins [162]. Conversely, human Ku70 binds hairpin RNA and double stranded DNA through two different sites [163], leaving open the possibility that Ku acts as a bridge (or even a recruiter) between TERC and DNA. We think that this function is different from the well-established association of Ku with telomerase through physical interaction with TERT [164]. Indeed, association between Ku and TERC has also been observed in HA5 (human embryonic kidney cells) and GM847 (human SV40-transformed ALT fibroblasts), which are proficient in TERC but are deficient in TERT [161]. Therefore, the specific link between TERC and Ku needs further study to understand its biological function and relevance. Furthermore, another question is raised by these interactions: does TERC associate with telomeres independently from telomerase? Usually the answer is no, as TERC needs TERT to localize to Cajal bodies (where telomerase is assembled) and to telomeres [165]. However, we think that this issue deserves further study.

Besides Ku, TERC also binds to the heterogeneous nuclear ribonucleoprotein A1 (hnRNPA1, [166]). Indeed, it has subsequently been demonstrated that TERC, DNA-PK (formed by Ku and DNA-PKcs) and hnRNPA1 form a complex, in which TERC activates DNA-PK to phosphorylate hnRNPA1 [167]. Moreover, it was later shown that DNA-PKcs-dependent phosphorylation of hnRNPA1 facilitates the RPA-to-POT1 switch and telomere capping after replication [168]. Thus, we hypothesize that TERC, through activation of DNA-PK and subsequent phosphorylation of hnRNPA1, removes RPA from telomeres, allowing telomere capping after replication (Figure 4).

Therefore, TERC, through its interaction with DNA-PK, seems to participate in telomere regulation in a manner that is functionally independent from TERT. Does TERC need TERT in any case to be localized at telomeres? If so, this telomeric function of TERC would be absent in telomerase-negative cells.

A possible answer to some of the questions raised above comes from a very recent (and brilliant) study that was published while we were finishing writing the present review. Raghunandan et al. [169], considering the newly found functions of TERC described in this section, started from the observation that TERC is absent in many ALT cells, TERC inhibits ATR ([149], see Section 4.1) and ALT cells display hypersensitivity to ATR inhibitors [170]. Using cell hybrids derived from ALT and telomerase-positive cells, they showed that TERC reduces phosphorylation of RPA at ALT telomeres by promoting the hnRNPA1- and DNA-PK-dependent depletion of RPA (thus confirming our hypothesis raised above). This causes a defective ATR checkpoint signalling at telomeres, thus impairing recruitment of RAD51, which is vital for the recombinogenic maintenance mechanism of ALT telomeres, and for increasing DNA damage signalling at telomeres. In this way, Raghunandan et al. [169] showed why loss of TERC expression is needed in ALT cells. However, although some ALT cell lines show no TERC expression, i.e., U2OS, KPD, NY (osteosarcoma cell lines) and immortalized fibroblasts (WI38-VA13, SUSM-1, KMST6, MDAH087), other ones show normal (AG11395 fibroblasts) or even high (SaOS-2 osteosarcoma, SK-LU-1 lung carcinoma, GM847 fibroblasts) levels of TERC. According to Raghunandan et al. [169], this contradiction is explained by the fact that TERC-expressing ALT cells downregulate NHP2 (a ribonucleoprotein associated with the H/ACA domain of TERC, see Section 2.4) through proteosomal degradation. This is quite surprising, since it has been previously known that NHP2 downregulation leads to TERC degradation [171]. Indeed, Raghunandan et al. [169] also found that NHP2, in telomerase-positive cells, causes downregulation of TERC. Moreover, these authors also found that ectopic overexpression of NHP2 in ALT cells caused downregulation of TERC (thus the opposite of what is found in telomerase positive cells). This complicated picture is explained by Raghunandan et al. [169] through the fact that NHP2-overexpressing ALT cells may experience adaptation mechanisms to cope with the simultaneous robust expressions of both NHP2 and TERC. In a similar manner, the authors justify the inverse correlation between NHP2 and TERC, which they found in ALT cells: these cells adapt to retained TERC expression through the downregulation of NHP2. Raghunandan et al. [169] found that this inverse correlation is present not only in their cell hybrids, but also in well-known ALT cells: U2OS, SaOS-2 and WI38-VA13 [169]. However, our analyses (although limited to three lines: U2OS, SaOS-2 and SKLU-1) from data on the Depmap portal (depmap.org) show that in ALT cells TERC expression is directly (and not inversely) correlated with NHP2 protein expression.

The need to downregulate NHP2 in ALT cells is because of its ability (found by [169]) to help the recruitment of 53BP1 (signalling DNA damage) to telomeres. Summing up, Raghunandan et al. [169] explained that TERC would be detrimental in ALT cells, impeding RAD51 activity at telomeres and causing NHP2-mediated DNA damage signalling. For this reason, many ALT cells have lost TERC expression, and those still retaining it downregulate NHP2 in order to hamper DNA damage signalling. However, we raised doubts about NHP2 downregulation in TERC-positive ALT cells and, interestingly, TERC-positive SaOS-2 and GM847 cells show higher levels of telomeric DNA damage signalling than TERC-negative U2OS cells [172,173]. We would argue that, in ALT cells, which are all p53-defective, persistent DNA damage signalling is not detrimental.

Despite this issue about the interplay between TERC and NHP2 in ALT cells (which needs further clarification), the study of Raghunandan et al. [169] confirms our hypothesis on the novel function of TERC as a partner of DNA-PK in the hnRNPA1-mediated removal of RPA at telomeres.

### 4.3. TERC and Mitochondria

**Figure 5:**
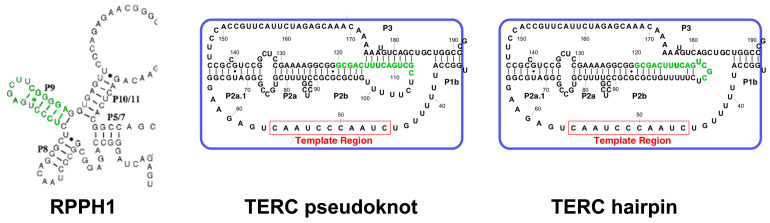
RNA structure and mitochondrial import. RPPH1 (RNA component of RNAse P, **left**) shows a stem-loop (in green) that has been experimentally proved to be necessary for mitochondrial import by PNPASE [176]. Cheng et al. [180] () found a similar sequence in TERC (in green) and considered it to have the same structure and role. The latter, however, is not a stem-loop, but is part of the pseudoknot, in the canonical conformation of TERC (**center**). In the alternative, temporary conformation of TERC (**right**) proposed by Denesyuk and Thirumalai [181], the sequence is part of a hairpin.

It has been demonstrated that mitochondria import a wide range of non-coding RNAs, including tRNAs, rRNAs, microRNAs, and long non-coding RNAs (lncRNAs) [174,175,176,177,178]. The import of most RNAs depends on a protein of the intermembrane space (IMS), polynucleotide phosphorylase (PNPASE) [176]. PNPASE is directly involved in regulating RNA import and could work as an import channel [176,179]. In particular, Wang et al. [176] found a stem-loop present both in RNAse P RNA (RPPH1) and MRP RNA (RMRP) responsible for their mitochondrial import. Cheng et al. [180], “looking for both sequence and structure similarity” to this stem-loop, “identified a non-coding RNA with a similar stem loop, the RNA component of human telomerase hTERC”. However, the sequence they found (CGCUGACUUUCAGCG, nucleotides 106–120) is not a stem-loop, but is part of the pseudoknot (Figure 5). Nevertheless, it was demonstrated in vitro that the pseudoknot structure of TERC is in equilibrium with a hairpin one (Figure 5) and it has been proposed that molecular crowding favors the first over the second [181]. Thus, it is possible that in the cytosol (where it is much more diluted than in the nucleus), TERC shows the hairpin conformation, and this could be recognized by PNPASE. However, this stem-loop is *different* from the one needed for RPPH1 mitochondrial import (Figure 5) and all these speculations need experimental evidence.

In any case, Cheng et al. [180] reported experimentally on the mitochondrial import of TERC, and discovered two different products of this RNA, wherein one of 195 nucleotides length called TERC-53, has been identified in the cytoplasm. The very selective cellular distribution of TERC-53 suggested that either there was a TERC processing activity in the cytosol, or that TERC was processed within mitochondria and then exported out into the cytosol. The authors, isolating different cellular fractions, identified the full-length TERC in the mitochondria, while TERC-53 was exclusively in the cytosol.

Therefore, the question about which enzyme cut TERC-53 arose. Liu et al. [182] previously identified a ribonuclease RNASET 2 in the IMS of mitochondria that degrades mitochondrial RNAs. RNASET2 is a ribonuclease that catalyzes the cleavage of RNAs in different cellular compartments [182,183,184]. Interestingly, the majority of TERC-53 was detected in the cytosol, and the cytosolic levels of TERC-53 were regulated by mitochondrial functions but had no direct effect on mitochondria, suggesting that this could function as a mitochondrial retrograde signal [180]. Therefore, the idea is that this process is a way of communication between mitochondria and other cellular compartments. However, it remains to be confirmed if TERC does not perform any function in the mitochondria or if there is a specific function of TERC within mitochondria and, if so, what that function is.

Zheng et al. [185] observed that cytosolic TERC-53 regulates cellular senescence. To investigate the function of cytosolic TERC-53, the authors stably overexpressed this RNA in human fetal fibroblast and observed a faster senescence (showed by β-galactosidase positive cells and increased p16 expression) in the TERC-53 overexpressing cell compared to the normal ones.

Previously, TERC has been shown to interact with GAPDH in the nucleus [186], and nuclear translocation is a key step for GAPDH to influence the gene expression regulation [186,187,188,189]. Translocation of GAPDH in the nucleus occurs when the cells are subject to different environmental stresses, and GAPDH acts in DNA repair, autophagy and cell death [190,191,192,193]. Zheng et al. [185] discovered that TERC-53 can interact with the cytosolic pool of GADPH and interferes with its nuclear localization, concluding that this may be the way in which TERC-53 induces cellular senescence. However, since GAPDH in the nucleus enhances both DNA repair and the p53-pathway, it is difficult to understand how its nuclear exclusion exerted by TERC-53 would lead to senescence.

Zheng et al. [185] also studied gene expression following the ectopic expression of TERC-53 or a TERC-53-antisense RNA. Apart from the fact that no senescence-associated genes (p16, p21 or p53) were upregulated, it is striking that the significantly modulated genes were *all* downregulated, and *in both cases* (i.e., both in cells with TERC-53 and with anti-TERC-53). Even for those genes whose expression was measured by qRT-PCR, the presence of anti-TERC-53 did not reverse the effects of TERC-53, but instead increased them. Therefore, interpretation of these data is quite difficult.

### 4.4. TERC-Derived Small RNA

**Figure 6:**
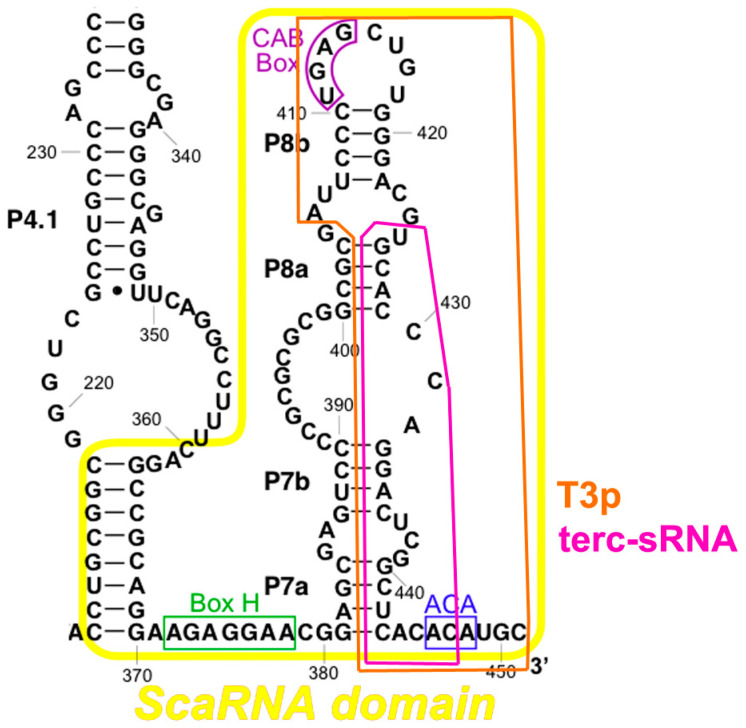
TERC derived sRNA. Fish et al. (2018) identified a small RNA corresponding to the 3′ end of TERC, from nucleotide 406 to 451 (T3p, in orange). Laudadio et al. (2019a) identified a small RNA, comprised in the range of T3p, called terc-sRNA (in purple). Both are within the ScaRNA domain (H/ACA box) of TERC (in yellow).

Recent studies have identified TERC as a source of small RNA (sRNA). Fish et al. [194] identified an sRNA corresponding to the last 45 nucleotides at the 3′ end of TERC, which they called T3p (Figure 6). This is highly enriched in breast cancer cells and exerts its pro-metastatic effects by increasing the expression of the genes *NUPR1* and *PANX2* and acting as an inhibitor of RNA-induced silencing complex (RISC) activity [194]. We would like to point out that the portion of TERC corresponding to T3p is exactly the one that binds to Ku (see Section 4.2.2).

Subsequently, Laudadio et al. [195] identified, in HeLa cells, an sRNA corresponding to a slightly smaller portion of T3p (Figure 6), and called it terc-sRNA. When it is overexpressed, it is sufficient to enhance telomerase activity, and analyses of sRNA-seq datasets showed that terc-sRNA is detected in primary human tissues and increases in tumors as compared to control tissues [195]. It has been proposed that terc-sRNA facilitates the association between TERC and Argonaute proteins, which, in turn, have recently been showed to be necessary for the proper assembly of the telomerase complex [196].

### 4.5. Translation of TERC?

Rubtsova et al. [197], starting from the fact that numerous lncRNAs have recently been found to contain short open reading frames [198], reported the discovery that TERC codes a 121 amino acid protein, which they called hTERP (human Telomerase RNA Protein). The corresponding 366-long putative open reading frame starts from nucleotide 176 of *TERC* and ends 90 nucleotides after the *TERC* gene. Thus, hTERP would be translated from a longer, immature form of TERC. This form has been reported in HeLa cells [199], although amounting to 1% of the normal 451 nt-long TERC.

#### 4.5.1. Prediction of TERC Protein-Coding Ability

In reality, this putative protein has been automatically identified since the first version of the human whole genome sequence (2005), and is still present as “hCG2044896” (accession # EAW78548.1) in the Celera-derived human genomic scaffold (accession # CH471052.2), but not in the human reference genome (assembly GRCh38.p13). However, the mere presence of an open reading frame (ORF) does not necessarily imply that it is translated.

One of the main methods used to elucidate this is the detection of evolutionary signatures characteristic of conserved coding regions, such as high frequencies of synonymous codon substitutions and conservative amino acid substitutions. One exemplary case is the newly identified protein SPAAR, whose coding sequence is comprised in the lncRNA gene *LINC00961* [198]. It can be easily viewed on the PhyloCSF (Phylogenetic Codon Substitution Frequencies) track on the UCSC Genome Browser that the putative hTERP-coding region is very poorly conserved. Indeed, it has not been included among new coding ORFs identified with this approach [200]. Moreover, van Heesch et al. [201] analyzed translatomes of human, mouse and rat hearts (and also human livers and kidneys), identifying hundreds of previously undetected microproteins, expressed from lncRNAs and circRNAs, but with no signs of hTERP. Furthermore, Secondo Ruiz-Orera and Albà [202] reported that mouse *TERC* gene does not contain an ORF. Thus, according to computational methods, TERC *does not* encode a protein.

#### 4.5.2. Evidence of Absence or Absence of Evidence?

The investigation of Rubtsova et al. [197] on the coding potential of TERC was suggested, as they wrote, by the fact that the literature reports that its transcript is present in the cytosol, is polyadenylated (such as mRNAs) and is associated with ribosomes. Presence in the cytosol is interesting, but could be explained by the newly identified functions of TERC shown above, like its shuttling into mitochondria. Concerning polyadenylation, this phenomenon is not restricted to mRNAs but is also part of the maturation/regulation process of many ncRNAs. Indeed, TERC is matured via a polyadenylation-dependent pathway that relies on the poly(A)-binding protein PABPN1 and the poly(A)-specific RNase PARN, whereas hTRAMP-dependent polyadenylation and exosome-mediated degradation function antagonistically to TERC maturation [203].

Finally, it is true that *some* studies employing ribosome-RNA-sequencing (Ribo-seq) reported the presence of TERC, but also that some studies reported its absence. This can be easily viewed on the GWIPS-viz track on the UCSC human genome (Figure S4 in Supplementary Material).

However, data from Ribo-seq should be interpreted with caution. This technique comprises the digestion of free RNA and then sequencing of the remaining RNA, assuming that this fraction is protected from digestion by its association with the ribosomes. However, ncRNAs that are associated with protein forming ribonucleoprotein complexes, such as TERC within telomerase, are also not digested and will therefore be present in Ribo-seq data. This issue has been investigated by Ingolia et al. [204], who succeeded in discriminating between true ribosome footprints and background RNA contained in non-ribosomal ribonucleoprotein complexes such as RNase P, vault RNP, and… telomerase. Indeed, it is interesting to note that most Ribo-seq data report the presence of the last part of the *TERC* gene, corresponding to the ScaRNA domain. This could represent TERC bound to dyskerin, NHP2 and TCAB1, but not to TERT (therefore with the ScaRNA domain protected from digestion, and the pseudknot and CR4/5 domains unprotected).

Finally, it should be noted that all Ribo-seq studies that report the presence of TERC show only the canonical (451 nucleotide-long) sequence, and there is no sign of the 90 nucleotides after the *TERC* gene reported by Rubtsova et al. [197] as part of the hTERP-coding region. In our view, these last data suggest that there is evidence of an absence of hTERP-coding mRNA in ribosomes.

#### 4.5.3. TERP

As explained above, computational methods indicate that *TERC* does not contain an ORF, its polyadenylated form is part of its ncRNA-specific maturation, and its elongated form is not present in ribosomes. Nonetheless, Rubtsova et al. [197], using HEK293T cells, reported the existence of hTERP by immunoblotting, immunofluorescence microscopy and mass spectroscopy, and experimental evidence is stronger than computational predictions. Through gain- and loss-of-function experiments, they showed that hTERP protects cells from apoptosis and participates in the processing of autophagosome. On the other hand, Brenner [205] did not confirm these functions, although she used another cellular model. Indeed, she demonstrated that TERC-ΔscaRNA, which disrupts the hTERP ORF, is sufficient to rescue TERC-KO human embryonic stem cells from doxorubicin-induced apoptosis.

All these interesting results, in our view, would need confirmation from other authors, also in order to elucidate the discrepancies between *TERC* gene predicted features and hTERP. Moreover, investigations employing cells from other mammalian species would give a clearer picture. The alignment of putative TERP in mammals (Figure S5 in Supplementary Material) shows very poor conservation, both in length and sequence. Length heterogeneity is seen between closely related species, such as human and macaque (121 vs. 53 aa), rabbit and pika (78 vs. 24), etc. In murines, the sequence does not start with methionine, most probably indicating absence of translation. The same is true for non-mammals and non-placental mammals. Thus, if the existence of hTERP will be confirmed, it will raise other questions. Is it a placental or primate novelty? Since only the first portion is conserved (in some species), which is its actual length? Moreover, Rubtsova et al. [197] raised the possibility that hTERP is processed to a smaller micropeptide. As we can see, this issue is still at the beginning of its study.

### 4.6. Cellular Effects

When using cellular or animal models in which TERC is silenced (or mutated), it is not easy to uncouple the telomerase-independent effects from the ones induced by inactivation of telomerase activity. One of the first studies on TERC [206] revealed that normal human skin fibroblasts (BJ cell line) transfected with antisense-TERC showed no effect for at least 38 doublings after transfection. Thus, it seems that TERC is dispensable in telomerase-negative fibroblasts under normal conditions. To this, we would add also that there are some cell lines that do not express TERC (because of the hypermethylation of its promoter [207]). These are U2OS (cancerous), and WI-38-VA13, KMST-6 and SUSM-1 (transformed fibroblasts). All these lines maintain their telomere length by a recombinogenic mechanism known as ALT (Alternative Lengthening of Telomeres) and, more importantly, like all ALT cells they are all p53-inactive, either because of over-expression of MDM2 or the presence of the SV40 antigen ([208]).

On the other hand, several articles reported the anti-apoptotic role of TERC. Many of the studies on gene expression cited previously (Section 4.1) observed apoptosis in cancer cells [142,143] and senescence in bone marrow stromal cells [146] after TERC depletion.

Gazzaniga and Blackburn [147] demonstrated an anti-apoptotic role of TERC in stimulated CD4 T cells. They demonstrated that overexpression of both wild-type and enzymatically inactive mutants of TERC (therefore independent of telomerase activity) protected from dexamethasone-induced apoptosis. Conversely, they found that silencing TERC induced Bim-mediated apoptosis after 12 days. None of these effects were seen in TERT-silenced cells. Moreover, TERC-silencing did not induce neither telomere damage nor shortening. Therefore, TERC anti-apoptotic properties were telomerase- and telomere-independent. Interestingly, mutant TERC with disrupted CR4/5 domain was ineffective in reducing apoptosis. Moreover, overexpression of TERT increased apoptosis, but not if it was coupled with a TERC mutant unable to bind TERT. However, the authors reported that TERT isoform β, which is catalytically inactive but binds to TERC, also did not increase apoptosis. Their interpretation was that TERT in a catalytically inactive form protects from apoptosis, and that binding of TERC per se does not affect its anti-apoptotic role. This latter deduction is quite difficult to understand for us. Summing up, the study of Gazzaniga and Blackburn [147] showed that, independently of telomerase, a portion of TERC, including the CR4/5 domain, is anti-apoptotic. This is in striking agreement with the studies on TERC-53, as the two regions overlap.

In order to investigate the consequences of TERC ablation in the absence of telomere dysfunction, Brenner [205] generated conditional TERC knock-out human embryonic stem cells (iTERC_KO hESCs) to uncouple TERC expression from telomerase activity. She found that deletion of TERC led to the widespread induction of apoptosis in the absence of short or dysfunctional telomeres. This phenotype was not recapitulated in conditional TERT knock-out hESCs and was prevented by expression of a TERC mutant RNA lacking the ScaRNA domain (ΔscaRNA). The conclusion drawn was that TERC has an essential function independent of the telomerase complex in hESCs. Moreover, she found that TERC is dispensable in somatic cells (thus, in agreement with [206]). Interestingly, TERC-53 was necessary and sufficient to prevent apoptosis in iTERC_KO hESCs. This result is different from that of Zheng et al. [185], who showed that TERC-53 overexpression induces senescence in fibroblasts (Section 4.2), but not necessarily in contradiction. First of all, apoptosis is different from senescence (actually, senescence is anti-apoptotic). Moreover, Zheng et al. [185] found that fibroblasts overexpressing TERC-53 do not senescence immediately, but reach senescence earlier than their normal counterparts. Curiously, they measured p16 and not p21 induction as a marker of senescence [185]. The latter is usually considered as a marker of replicative (i.e., due to telomere shortening) senescence, while p16 is best described as a marker of oncogene-induced senescence [209]. This TERC-53-induced p16 expression, coupled with the cell type-specific effects of TERC silencing/overexpression (Table 1), tempts us to draw a parallel between TERC and oncogenes: in differentiated, slowly proliferating cells (such as fibroblasts) TERC is not necessary, and its overexpression leads to senescence, while in stem, tumor and fast proliferating cells, TERC is necessary and its absence leads to apoptosis. Further studies on TERC silencing/overexpression in different cell types could elucidate this issue. In particular, telomerase-negative (i.e., ALT) immortal cells with high (SaOS-2, GM847), normal (AG11395) and no (U2OS, WI-38-VA13) TERC expression could represent interesting models.

## 5. The Network of TERT and TERC Functions and Interactions

**Figure 7:**
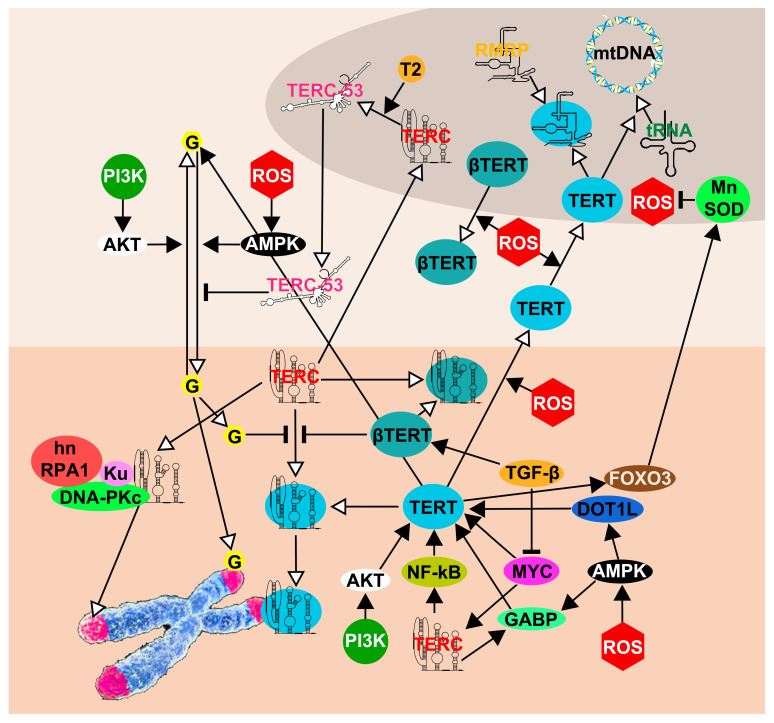
TERT and TERC functions and interactions. PI3K/AKT increase TERT transcription and lead to increased telomerase activity. Moreover, ROS/AMPK increase TERT transcription, but concurrently translocate GAPDH into the nucleus (blocking TERT/TERC) assembly, and translocate TERT to mitochondria (via cytosol), therefore decreasing telomerase activity and increasing non-telomeric functions of TERT. Among these, there is an increase in MnSOD (via FOXO3), which detoxifies ROS, and other mitochondrial functions not yet fully elucidated. G (in yellow hexagons): GAPDH; T2 (in orange circle): RNASET2. For ncRNAs (TERC, TERC-53 and RMRP), secondary structures are shown. Full arrows indicate stimulation, empty arrows indicate translocation.

A review of the factors that regulate the transcription of *TERT* and *TERC* is beyond the scope of this article. Nonetheless, we will briefly deal with some of them, since some proteins whose transcription/localization is influenced by TERT are regulators of *TERC*, and vice versa (Figure 7).

As reported above, TERC enhances the expression of *NF-κB* [145], which stimulates *TERT* [86]. MYC is a well-known stimulator of *TERT* [89], but it has also been shown to increase *TERC* expression [211]. Furthermore, the PI3K/AKT axis increases TERT transcription [212], and we will show that it is also involved in other pathways indirectly linked with TERT and TERC. Moreover, a recent paper showed a positive feedback regulation between TERC and the PI3K-AKT pathway (independent of telomerase activity) in human fibroblasts [213]. Conversely, the pro-differentiation growth inhibiting factor TGF-β1 not only represses c-Myc (and thus, indirectly TERC and TERT), but also changes the balance between TERT isoforms, favoring isoform β over full length TERT [214]. Isoform β has been proposed to compete with full length TERT for TERC binding (Figure 7), thus decreasing telomerase assembly and activity (see Section 3.5).

The AMP-activated protein kinase (AMPK) signaling pathway also shows several interactions with TERT and TERC. Jo et al. [215] observed that AMPK, through stimulation of *MYC*, increases the expression of *TERT*. Moreover, using the AMPK inducers Metformin and AICAR (5-Aminoimidazole-4-carboxamide ribonucleotide) in endothelial cells, Karnewar et al. [216] showed that AMPK enhances the transcription of the deacetylase *SIRT1*, which leads (through activation of *DOT1L*) to increased methylation levels of histone H3K79. Interestingly, they observed that AMPK-induced H3K79me enhances the transcription of *PGC-1α*, increasing mitogenesis, and *SIRT3*, which is thought to be a direct inducer of *TERT* [216]. Furthermore, McKinney et al. [217] showed that AMPK inhibition decreases transcription of both subunits of the tetrameric GA-binding protein complex (GABPA and GABPB1), reducing TERT expression and telomerase activity in a TERT promoter mutation dependent manner. Thus, we can sum up that AMPK induces TERT expression both via SIRT1, DOT1L, SIRT3 and via GABPA, GABPB1 (Figure 7). It is interesting to note that GABPA and GABPB1 expression is also upregulated by TERC (see Section 4.1), showing an additional pathway by which TERT and TERC are interlinked (Figure 7).

Another interesting interactor is GAPDH, since (as reported above, see Section 3.1.6) it has been showed to be upregulated by TERT [99] and it interacts with TERC in the nucleus, hampering the assembly of the telomerase complex [186]. Moreover, Zheng et al., [185] reported that TERC-53 interferes with the nuclear localization of GAPDH (see Section 4.3). This enzyme is mainly used in glycolysis (Figure 7), a pathway (as described above) that seems to be upregulated by TERC [144]. Moreover, upon activation of AMPK, GAPDH enters the nucleus, whereas following PI3K-Akt-mediated mitogenic stimuli, it exits from the nucleus into the cytosol [218]. Inside the nucleus, besides interacting with TERC, GAPDH binds to both single- and double-stranded telomeric DNA and protects it from degradation [219,220]. Moreover, Pariona-Llanos et al. [221] showed that in replicative epimastigotes of *Trypanosoma cruzi* GAPDH also binds telomeres, whereas in non-proliferative trypomastigote forms, which show higher NAD+ concentration, GAPDH was absent from telomeres (concluding that NAD+ reduces GAPDH-telomere interaction).

The connections between TERT, TERC and glycolysis are interesting, in our opinion, in the light of a stemness state. Glycolysis is a metabolic feature of cancer cells, pluripotent embryonic stem cells, activated T-lymphocytes, macrophages, and endothelial cells during angiogenesis [222]. Differentiation, on the other hand, increases mitochondrial abundance and oxidative metabolism [223,224]. Conversely, induced pluripotent stem cells upregulate glycolytic enzymes and downregulate electron transport chain subunits, converting somatic oxidative metabolism into a glycolytic flux-dependent and mitochondria-independent state that underlies pluripotency induction [224]. Moreover, Ahmed et al. [115] found that TERT-overexpressing fibroblasts show a lower mitochondrial mass compared to normal fibroblasts, a result that we interpret as further proof that TERT causes a shift toward glycolysis. Incidentally, it should be added that such a shift toward glycolysis leads to a reduction in ROS production [225,226].

Whereas the decrease of ROS levels due to TERT- and TERC-induced glycolysis is a deduction of ours, there are experimental studies showing TERT involvement in ROS detoxification. In particular, TERT increases the mitochondrial levels of manganese superoxide dismutase (MnSOD) via FoxO3 (see Section 3.4), thus leading to a decrease in ROS (Figure 7).

Oxidative stress also causes TERT’s exit from the nucleus and its entry in the mitochondria (see Section 3.3.1), and at the same time expulsion of the TERT isoform β from the latter (see Section 3.5). TERT functions inside mitochondria need to be elucidated, and so far interactions with RMRP (see Section 3.2), mitochondrial DNA and tRNA (see Section 3.3.2) have been proposed (Figure 7).

Moreover, TERC has been showed to be able to enter mitochondria (see Section 4.3), where it seems to be processed by RNAseT2 into TERC-53 (Figure 7). Cellular effects of TERC-53 are dubious and/or contradictory (see Section 4.3 and Section 4.6) and the only mechanistic molecular interaction so far proposed is its sequestration of GAPDH in the cytosol (Figure 7).

Finally, we would like to evidence the telomeric functions of TERC and TERT: the first (obviously) is the formation of the telomerase complex (with TERT) that elongates telomeres; moreover, GAPDH (which protects telomeric DNA) is upregulated by TERT and its nuclear entry is blocked by TERC-53 (Figure 7); finally, TERC forms a complex with Ku and DNA-PKc that performs hnRNPA1-mediated removal of RPA at telomeres (see Section 4.2.2).

Summing up, we would propose two major pathways emerging from this complicated scheme. In the first, mitogenic stimuli (via PI3K/AKT) increase TERT expression and lead to an increase of telomerase formation and activity. In the second, oxidative stress also leads to increased TERT expression; however, in this case this is coupled with other phenomena (i.e., TERT exit from the nucleus, GAPDH entry) that decrease telomerase assembly, thus making non-telomerase function of TERT and TERC prevail (Figure 7).

## 6. Open Questions for Future Research

Does TERC associate directly with telomeres in absence of TERT? If so, what are its function in this context? Performing enChIP (engineered DNA-binding molecule-mediated chromatin immunoprecipitation)-RT-PCR, Fujita et al. [227] detected TERC at telomeres in mouse hematopoietic cells, which are telomerase-positive and thus could be ascribed to the presence of the telomerase complex bound to telomeres. However, among many telomere-bound proteins found by Fujita et al. [227] through enChIP-mass spectrometry, TERT is lacking. Furthermore, Déjardin and Kingston [228] failed to identify TERT (and dyskerin) at HeLa telomeres, while they found all the telomeric proteins (TRF1, TRF2, POT1, etc.). They explained it by the fact that TERT does not represent a constitutive component of telomere chromatin and so would not be expected to be telomere-bound in a significant percentage in non-synchronized cells. The fact that TERT cannot be found, but TERC is telomere-bound (in the case of [227] this discrepancy happened in the same cells), suggests to us that TERC may also be present at telomeres when TERT is not. Performing enChIP-RT-PCR in TERC-positive, TERT-negative cells (e.g., fibroblasts) could answer this question.

Another issue that needs further study is the cell-type specific effects of TERC silencing and overexpression (see Section 4.6). Using appropriate cellular models and a larger panel of cell type will give important information. The same can be said for the newly found interactions of TERC with other proteins (see Section 4.2). Moreover, the controversial ability of TERC to be translated into TERP (see Section 4.6) needs to be clarified, both in human and non-human cells.

Moreover, a topic that seems to be almost absent in the studies on TERC is the role of its H/ACA partner proteins (dyskerin, NOP10, NHP2, GAR1 and TCAB1): when TERC exerts its non-canonical activities, is it bound to any of them?

Regarding TERT, the amount of research dealing with its extra-telomerase function is much bigger than that of TERC. Nonetheless, many results remain contradictory or dubious. Above all, there is a need for appropriate cellular models that allow the disentangling of the effects due to TERT silencing and overexpression from those due to telomerase inhibition and activation. For example, ectopic expression of TERT in fibroblasts leads to reactivation of telomerase activity (which is absent in normal fibroblasts), and silencing of TERT in cancer cells leads to inhibition of telomerase activity. Appropriate cellular models for the study of the effects of TERT ectopic expression would be TERC-negative cells, such as U2OS, WI-38-VA13 (both used by [97], see Section 3.1.6), KMST-6 and SUSM-1. On the other hand, in order to study the effects of TERT silencing, it would be wise to use TERT-positive, TERC-negative cell lines, but to our knowledge, these seem to be absent. Nonetheless, this problem could be circumvented by the use of murine fibroblasts (which normally display TERT expression, contrarily to human ones) obtained by TERC-KO mice (mTR^−/−^).

Furthermore, the link between TERT and RMRP (see Section 3.2) needs further investigation, since the RNA-dependent RNA polymerase activity of TERT supported by Maida et al. [100] is contradicted by the results of other authors [96,101,229].

The interesting field of investigation on TERT activities in mitochondria still shows some open questions: what is the real actual activity of TERT in these organelles? Does it interact directly with mitochondrial DNA? How does it participate (if it really does) in mitochondrial DNA replication? Besides these questions, we also think that the study of TERT interactions with mitochondria will be greatly improved if the different TERT isoforms are taken into consideration.

Finally, it should be considered that early research suffered from a lack of reliable antibodies that immunoprecipitate endogenous TERT specifically, preventing, for example, a direct assessment of the global genome occupancy signature for endogenous TERT. It has also been shown that many commercial antibodies actually detect nucleolin, which has the same molecular weight as TERT, causing western blots to be unreliable [230]. Therefore, many results of old studies should be considered with caution, and it could be beneficial to repeat these studies again with new, more specific antibodies.

## 7. Conclusions

With the progress of techniques in the fields of molecular biology, proteomics, RNA biology and epigenetics, more and more functions of TERT and TERC have come to light. Although many observations remain controversial or dubious, it seems clear that these two components of the telomerase holoenzyme have many extra-telomeric functions. Of the two, TERT is by far the most studied and its role in the regulation of many genes seems ascertained, also because mechanistic findings have been provided. Many other functions seem to give TERT a role in the response to oxidative stress and, in particular, in mitochondrial protection. However, it remains to elucidate the importance of this role, since many human somatic cells are devoid of TERT, such as fibroblasts, which in turn are the most resistant to oxidative stress (as can also be seen from the ease with which these can be grown in vitro).

Further functions of TERT, as some functions of TERC, remain dubious or even unrealistic. We suggest that researchers directly read the articles that investigated these functions and form their own opinion on them, rather than rely on uncritical reviews that disseminate phenomena that still need confirmation as certainties. Above all, there is a need for confirmation on many issues, i.e., to repeat studies already undertaken and verify the proposed mechanisms.

## Figures and Tables

**Table 1:** Effects of TERC silencing and overexpression in different cell types. BMSCs: bone marrow stromal cells; ESC: Embryonic stem cells; DM: Dexamethasone; DOX: Doxorubicin.

Cell Type	TERT Expression	TERC Silencing	TERC Overexpression	TERC-53 Overexpression
Human Fibroblasts	No	No effects [205,206]		
Human Fibroblasts	No			Faster senescence [185]
Human BMSCs	No [210]	Senescence [146]		
Human CD4 T cells	Yes [147]	Apoptosis [147]	Reduction of DM-induced apoptosis [147]	
Human ESCs	Yes [205]	Apoptosis [205]		Reduction of DOX-induced apoptosis [205]
HeLa and HCT116 cancer cells	Yes	Apoptosis [142,143]		

## Data Availability

All data are available in the main text and Supplementary Material.

## Supplementary Materials

The supporting information can be downloaded at: https://www.mdpi.com/article/10.3390/ijms232315189/s1.

## References

[1] Greider C.W., Blackburn E.H. (1985). Identification of a Specific Telomere Terminal Transferase Activity in Tetrahymena Extracts. Cell.

[2] Olovnikov A.M. (1971). Principle of Marginotomy in Template Synthesis of Polynucleotides. Dokl. Akad. Nauk SSSR.

[3] Shay J.W., Wright W.E. (2002). Telomerase: A Target for Cancer Therapeutics. Cancer Cell.

[4] Morrison S.J., Prowse K.R., Ho P., Weissman I.L. (1996). Telomerase Activity in Hematopoietic Cells Is Associated with Self-Renewal Potential. Immunity.

[5] Härle-Bachor C., Boukamp P. (1996). Telomerase Activity in the Regenerative Basal Layer of the Epidermis Inhuman Skin and in Immortal and Carcinoma-Derived Skin Keratinocytes. Proc. Natl. Acad. Sci. USA.

[6] Colitz C.M.H., Davidson M.G., McGahan M.C. (1999). Telomerase Activity in Lens Epithelial Cells of Normal and Cataractous Lenses. Exp. Eye Res..

[7] Rosen J., Jakobs P., Ale-Agha N., Altschmied J., Haendeler J. (2020). Non-Canonical Functions of Telomerase Reverse Transcriptase - Impact on Redox Homeostasis. Redox Biol..

[8] Gould S.J. (1998). Leonardo’s Mountain of Clams and the Diet of Worms: Essays on Natural History.

[9] Liang L., Zhong Z., Rousseau R. (2014). Scientists’ Referencing (Mis)Behavior Revealed by the Dissemination Network of Referencing Errors. Scientometrics.

[10] Udroiu I., Russo V., Persichini T., Colasanti M., Sgura A. (2017). Telomeres and Telomerase in Basal Metazoa. Invertebr. Surviv. J..

[11] Autexier C., Lue N.F. (2006). The Structure and Function of Telomerase Reverse Transcriptase. Annu. Rev. Biochem..

[12] Greider C.W., Blackburn E.H. (1989). A Telomeric Sequence in the RNA of Tetrahymena Telomerase Required for Telomere Repeat Synthesis. Nature.

[13] Venteicher A.S., Artandi S.E. (2009). TCAB1: Driving Telomerase to Cajal Bodies. Cell Cycle.

[14] Hockemeyer D., Collins K. (2015). Control of Telomerase Action at Human Telomeres. Nat. Struct. Mol. Biol..

[15] Zhang Q., Kim N.K., Feigon J. (2011). Architecture of Human Telomerase RNA. Proc. Natl. Acad. Sci. USA.

[16] Morin G.B. (1989). The Human Telomere Terminal Transferase Enzyme Is a Ribonucleoprotein That Synthesizes TTAGGG Repeats. Cell.

[17] Greider C.W. (1991). Telomerase Is Processive. Mol. Cell. Biol..

[18] Weise J.M., Günes C. (2009). Differential Regulation of Human and Mouse Telomerase Reverse Transcriptase (TERT) Promoter Activity during Testis Development. Mol. Reprod. Dev..

[19] Avilion A.A., Piatyszek M.A., Gupta J., Shay J.W., Bacchetti S., Greider C.W. (1996). Human Telomerase RNA and Telomerase Activity in Immortal Cell Lines and Tumor Tissues. Cancer Res..

[20] Castle J.C., Armour C.D., Löwer M., Haynor D., Biery M., Bouzek H., Chen R., Jackson S., Johnson J.M., Rohl C.A. (2010). Digital Genome-Wide NcRNA Expression, Including SnoRNAs, across 11 Human Tissues Using PolyA-Neutral Amplification. PLoS One.

[21] Hartmann N., Reichwald K., Lechel A., Graf M., Kirschner J., Dorn A., Terzibasi E., Wellner J., Platzer M., Rudolph K.L. (2009). Telomeres Shorten While Tert Expression Increases during Ageing of the Short-Lived Fish Nothobranchius Furzeri. Mech. Ageing Dev..

[22] Agarwal S., Loh Y.H., McLoughlin E.M., Huang J., Park I.H., Miller J.D., Huo H., Okuka M., Dos Reis R.M., Loewer S. (2010). Telomere Elongation in Induced Pluripotent Stem Cells from Dyskeratosis Congenita Patients. Nature.

[23] Weinrich S.L., Pruzan R., Ma L., Ouellette M., Tesmer V.M., Holt S.E., Bodnar A.G., Lichtsteiner S., Kim N.W., Trager J.B. (1997). Reconstitution of Human Telomerase with the Template RNA Component HTR and the Catalytic Protein Subunit HTRT. Nat. Genet..

[24] Miller M.C., Liu J.K., Collins K. (2000). Template Definition by Tetrahymena Telomerase Reverse Transcriptase. EMBO J..

[25] Moriarty T.J., Huard S., Dupuis S., Autexier C. (2002). Functional Multimerization of Human Telomerase Requires an RNA Interaction Domain in the N Terminus of the Catalytic Subunit. Mol. Cell. Biol..

[26] Sealey D.C.F., Zheng L., Taboski M.A.S., Cruickshank J., Ikura M., Harrington L.A. (2010). The N-Terminus of HTERT Contains a DNA-Binding Domain and Is Required for Telomerase Activity and Cellular Immortalization. Nucleic Acids Res..

[27] Lai C.K., Mitchell J.R., Collins K. (2001). RNA Binding Domain of Telomerase Reverse Transcriptase. Mol. Cell. Biol..

[28] Moriarty T.J., Marie-Egyptienne D.T., Autexier C. (2004). Functional Organization of Repeat Addition Processivity and DNA Synthesis Determinants in the Human Telomerase Multimer. Mol. Cell. Biol..

[29] Mitchell J.R., Collins K. (2000). Human Telomerase Activation Requires Two Independent Interactions between Telomerase RNA and Telomerase Reverse Transcriptase. Mol. Cell.

[30] Friedman K.L., Cech T.R. (1999). Essential Functions of Amino-Terminal Domains in the Yeast Telomerase Catalytic Subunit Revealed by Selection for Viable Mutants. Genes Dev..

[31] Willers H., McCarthy E.E., Wu B., Wunsch H., Tang W., Taghian D.G., Xia F., Powell S.N. (2000). Dissociation of P53-Mediated Suppression of Homologous Recombination from G1/S Cell Cycle Checkpoint Control. Oncogene.

[32] Beattie T.L., Zhou W., Robinson M.O., Harrington L. (1998). Reconstitution of Human Telomerase Activity in Vitro. Curr. Biol..

[33] Nakamura T.M., Morin G.B., Chapman K.B., Weinrich S.L., Andrews W.H., Lingner J., Harley C.B., Cech T.R. (1997). Telomerase Catalytic Subunit Homologs from Fission Yeast and Human. Science.

[34] Bosoy D., Lue N.F. (2001). Functional Analysis of Conserved Residues in the Putative “Finger” Domain of Telomerase Reverse Transcriptase. J. Biol. Chem..

[35] Xie M., Podlevsky J.D., Qi X., Bley C.J., Chen J.J.L. (2010). A Novel Motif in Telomerase Reverse Transcriptase Regulates Telomere Repeat Addition Rate and Processivity. Nucleic Acids Res..

[36] Gillis A.J., Schuller A.P., Skordalakes E. (2008). Structure of the Tribolium Castaneum Telomerase Catalytic Subunit TERT. Nature.

[37] Seimiya H., Sawada H., Muramatsu Y., Shimizu M., Ohko K., Yamane K., Tsuruo T. (2000). Involvement of 14-3-3 Proteins in Nuclear Localization of Telomerase. EMBO J..

[38] Haendeler J., Hoffmann J., Brandes R.P., Zeiher A.M., Dimmeler S. (2003). Hydrogen Peroxide Triggers Nuclear Export of Telomerase Reverse Transcriptase via Src Kinase Family-Dependent Phosphorylation of Tyrosine 707. Mol. Cell. Biol..

[39] Ye A.J., Romero D.P. (2002). Phylogenetic Relationships amongst Tetrahymenine Ciliates Inferred by a Comparison of Telomerase RNAs. Int. J. Syst. Evol. Microbiol..

[40] Chakrabarti K., Pearson M., Grate L., Sterne-Weiler T., Deans J., Donohue J.P., Ares M. (2007). Structural RNAs of Known and Unknown Function Identified in Malaria Parasites by Comparative Genomics and RNA Analysis. RNA.

[41] Song J., Logeswaran D., Castillo-González C., Li Y., Bose S., Aklilu B.B., Ma Z., Polkhovskiy A., Chen J.J.L., Shippen D.E. (2019). The Conserved Structure of Plant Telomerase RNA Provides the Missing Link for an Evolutionary Pathway from Ciliates to Humans. Proc. Natl. Acad. Sci. USA.

[42] Qi X., Li Y., Honda S., Hoffmann S., Marz M., Mosig A., Podlevsky J.D., Stadler P.F., Selker E.U., Chen J.J.L. (2013). The Common Ancestral Core of Vertebrate and Fungal Telomerase RNAs. Nucleic Acids Res..

[43] Logeswaran D., Li Y., Podlevsky J.D., Chen J.J.L. (2021). Monophyletic Origin and Divergent Evolution of Animal Telomerase RNA. Mol. Biol. Evol..

[44] Podlevsky J.D., Chen J.J.L. (2016). Evolutionary Perspectives of Telomerase RNA Structure and Function. RNA Biol..

[45] Chen J.L., Blasco M.A., Greider C.W. (2000). Secondary Structure of Vertebrate Telomerase RNA. Cell.

[46] Theimer C.A., Feigon J. (2006). Structure and Function of Telomerase RNA. Curr. Opin. Struct. Biol..

[47] Chen J.L., Greider C.W. (2003). Template Boundary Definition in Mammalian Telomerase. Genes Dev..

[48] Hossain S., Singh S., Lue N.F. (2002). Functional Analysis of the C-Terminal Extension of Telomerase Reverse Transcriptase. A Putative “Thumb” Domain. J. Biol. Chem..

[49] Förstemann K., Lingner J. (2005). Telomerase Limits the Extent of Base Pairing between Template RNA and Telomeric DNA. EMBO Rep..

[50] Brown Y., Abraham M., Pearl S., Kabaha M.M., Elboher E., Tzfati Y. (2007). A Critical Three-Way Junction Is Conserved in Budding Yeast and Vertebrate Telomerase RNAs. Nucleic Acids Res..

[51] Blackburn E.H., Collins K. (2011). Telomerase: An RNP Enzyme Synthesizes DNA. Cold Spring Harb. Perspect. Biol..

[52] Huang J., Brown A.F., Wu J., Xue J., Bley C.J., Rand D.P., Wu L., Zhang R., Chen J.J.L., Lei M. (2014). Structural Basis for Protein-RNA Recognition in Telomerase. Nat. Struct. Mol. Biol..

[53] Mitchell J.R., Cheng J., Collins K. (1999). A Box H/ACA Small Nucleolar RNA-like Domain at the Human Telomerase RNA 3’ End. Mol. Cell. Biol..

[54] Vulliamy T.J., Marrone A., Knight S.W., Walne A., Mason P.J., Dokal I. (2006). Mutations in Dyskeratosis Congenita: Their Impact on Telomere Length and the Diversity of Clinical Presentation. Blood.

[55] Li H. (2008). Unveiling Substrate RNA Binding to H/ACA RNPs: One Side Fits All. Curr. Opin. Struct. Biol..

[56] Reichow S.L., Hamma T., Ferré-D’Amaré A.R., Varani G. (2007). The Structure and Function of Small Nucleolar Ribonucleoproteins. Nucleic Acids Res..

[57] Cristofari G., Adolf E., Reichenbach P., Sikora K., Terns R.M., Terns M.P., Lingner J. (2007). Human Telomerase RNA Accumulation in Cajal Bodies Facilitates Telomerase Recruitment to Telomeres and Telomere Elongation. Mol. Cell.

[58] Theimer C.A., Jády B.E., Chim N., Richard P., Breece K.E., Kiss T., Feigon J. (2007). Structural and Functional Characterization of Human Telomerase RNA Processing and Cajal Body Localization Signals. Mol. Cell.

[59] Kiss T., Fayet-Lebaron E., Jády B.E. (2010). Box H/ACA Small Ribonucleoproteins. Mol. Cell.

[60] Egan E.D., Collins K. (2010). Specificity and Stoichiometry of Subunit Interactions in the Human Telomerase Holoenzyme Assembled in Vivo. Mol. Cell. Biol..

[61] Lafontaine D.L.J., Bousquet-Antonelli C., Henry Y., Caizergues-Ferrer M., Tollervey D. (1998). The Box H + ACA SnoRNAs Carry Cbf5p, the Putative RRNA Pseudouridine Synthase. Genes Dev..

[62] Ganot P., Bortolin M.L., Kiss T. (1997). Site-Specific Pseudouridine Formation in Preribosomal RNA Is Guided by Small Nucleolar RNAs. Cell.

[63] Kiss A.M., Jády B.E., Darzacq X., Verheggen C., Bertrand E., Kiss T. (2002). A Cajal Body-Specific Pseudouridylation Guide RNA Is Composed of Two Box H/ACA SnoRNA-like Domains. Nucleic Acids Res..

[64] Richard P., Darzacq X., Bertrand E., Jády B.E., Verheggen C., Kiss T. (2003). A Common Sequence Motif Determines the Cajal Body-Specific Localization of Box H/ACA ScaRNAs. EMBO J..

[65] Wang C., Meier U.T. (2004). Architecture and Assembly of Mammalian H/ACA Small Nucleolar and Telomerase Ribonucleoproteins. EMBO J..

[66] Grozdanov P.N., Roy S., Kittur N., Meier U.T. (2009). SHQ1 Is Required Prior to NAF1 for Assembly of H/ACA Small Nucleolar and Telomerase RNPs. RNA.

[67] Girard J.P., Lehtonen H., Caizergues-Ferrer M., Amalric F., Tollervey D., Lapeyre B. (1992). GAR1 Is an Essential Small Nucleolar RNP Protein Required for Pre-RRNA Processing in Yeast. EMBO J..

[68] Maiorano D., Brimage L.J.E., Leroy D., Kearsey S.E. (1999). Functional Conservation and Cell Cycle Localization of the Nhp2 Core Component of H + ACA SnoRNPs in Fission and Budding Yeasts. Exp. Cell Res..

[69] Venteicher A.S., Abreu E.B., Meng Z., McCann K.E., Terns R.M., Veenstra T.D., Terns M.P., Artandi S.E. (2009). A Human Telomerase Holoenzyme Protein Required for Cajal Body Localization and Telomere Synthesis. Science.

[70] Freund A., Zhong F.L., Venteicher A.S., Meng Z., Veenstra T.D., Frydman J., Artandi S.E. (2014). Proteostatic Control of Telomerase Function through TRiC-Mediated Folding of TCAB1. Cell.

[71] Flint J., Craddock C.F., Villegas A., Bentley D.P., Williams H.J., Galanello R., Cao A., Wood W.G., Ayyub H., Higgs D.R. (1994). Healing of Broken Human Chromosomes by the Addition of Telomeric Repeats. Am. J. Hum. Genet..

[72] Nergadze S.G., Santagostino M.A., Salzano A., Mondello C., Giulotto E. (2007). Contribution of Telomerase RNA Retrotranscription to DNA Double-Strand Break Repair during Mammalian Genome Evolution. Genome Biol..

[73] Nandakumar J., Cech T.R. (2013). Finding the End: Recruitment of Telomerase to Telomeres. Nat. Rev. Mol. Cell Biol..

[74] Furuya T., Morgan R., Berger C.S., Sandberg A.A. (1993). Presence of Telomeric Sequences on Deleted Chromosomes and Their Absence on Double Minutes in Cell Line HL-60. Cancer Genet. Cytogenet..

[75] Meltzer P.S., Guan X.Y., Trent J.M. (1993). Telomere Capture Stabilizes Chromosome Breakage. Nat. Genet. 1993 43.

[76] McClintock B. (1941). The Stability of Broken Ends of Chromosomes in Zea Mays. Genetics.

[77] Q F., M Y. (1996). New Telomere Formation Coupled with Site-Specific Chromosome Breakage in Tetrahymena Thermophila. Mol. Cell. Biol..

[78] Matsumoto T., Fukui K., Niwa O., Sugawara N., Szostak J.W., Yanagida M. (1987). Identification of Healed Terminal DNA Fragments in Linear Minichromosomes of Schizosaccharomyces Pombe. Mol. Cell. Biol..

[79] Kramer K.M., Haber J.E. (1993). New Telomeres in Yeast Are Initiated with a Highly Selected Subset of TG1-3 Repeats. Genes Dev..

[80] Wang S.S., Zakian V.A. (1990). Telomere-Telomere Recombination Provides an Express Pathway for Telomere Acquisition. Nature.

[81] Zhang J.M., Yadav T., Ouyang J., Lan L., Zou L. (2019). Alternative Lengthening of Telomeres through Two Distinct Break-Induced Replication Pathways. Cell Rep..

[82] Hong J., Lee J.H., Chung I.K. (2016). Telomerase Activates Transcription of Cyclin D1 Gene through an Interaction with NOL1. J. Cell Sci..

[83] Choi J., Southworth L.K., Sarin K.Y., Venteicher A.S., Ma W., Chang W., Cheung P., Jun S., Artandi M.K., Shah N. (2008). TERT Promotes Epithelial Proliferation through Transcriptional Control of a Myc- and Wnt-Related Developmental Program. PLoS Genet..

[84] Ding D., Xi P., Zhou J., Wang M., Cong Y.S. (2013). Human Telomerase Reverse Transcriptase Regulates MMP Expression Independently of Telomerase Activity via NF-ΚB-Dependent Transcription. FASEB J..

[85] Yin L., Hubbard A.K., Giardina C. (2000). NF-Kappa B Regulates Transcription of the Mouse Telomerase Catalytic Subunit. J. Biol. Chem..

[86] Sinha-Datta U., Horikawa I., Michishita E., Datta A., Sigler-Nicot J.C., Brown M., Kazanji M., Barrett J.C., Nicot C. (2004). Transcriptional Activation of HTERT through the NF-KappaB Pathway in HTLV-I-Transformed Cells. Blood.

[87] Ghosh A., Saginc G., Leow S.C., Khattar E., Shin E.M., Yan T.D., Wong M., Zhang Z., Li G., Sung W.K. (2012). Telomerase Directly Regulates NF-ΚB-Dependent Transcription. Nat. Cell Biol..

[88] Clevers H., Nusse R. (2012). Wnt/β-Catenin Signaling and Disease. Cell.

[89] Park J.I., Venteicher A.S., Hong J.Y., Choi J., Jun S., Shkreli M., Chang W., Meng Z., Cheung P., Ji H. (2009). Telomerase Modulates Wnt Signalling by Association with Target Gene Chromatin. Nature.

[90] Liu Z., Li Q., Li K., Chen L., Li W., Hou M., Liu T., Yang J., Lindvall C., Björkholm M. (2013). Telomerase Reverse Transcriptase Promotes Epithelial-Mesenchymal Transition and Stem Cell-like Traits in Cancer Cells. Oncogene.

[91] Sherr C.J., Roberts J.M. (2004). Living with or without Cyclins and Cyclin-Dependent Kinases. Genes Dev..

[92] Farwell D.G., Shera K.A., Koop J.I., Bonnet G.A., Matthews C.P., Reuther G.W., Coltrera M.D., McDougall J.K., Klingelhutz A.J. (2000). Genetic and Epigenetic Changes in Human Epithelial Cells Immortalized by Telomerase. Am. J. Pathol..

[93] Xiang H., Wang J., Mao Y., Wan-Cheng Li D., Liu M., Reddy V.N. (2002). Human Telomerase Accelerates Growth of Lens Epithelial Cells through Regulation of the Genes Mediating RB/E2F Pathway. Oncogene.

[94] Yang C., Przyborski S., Cooke M.J., Zhang X., Stewart R., Anyfantis G., Atkinson S.P., Saretzki G., Armstrong L., Lako M. (2008). A Key Role for Telomerase Reverse Transcriptase Unit in Modulating Human Embryonic Stem Cell Proliferation, Cell Cycle Dynamics, and in Vitro Differentiation. Stem Cells.

[95] Gonzalez O.G., Assfalg R., Koch S., Schelling A., Meena J.K., Kraus J., Lechel A., Katz S.F., Benes V., Scharffetter-Kochanek K. (2014). Telomerase Stimulates Ribosomal DNA Transcription under Hyperproliferative Conditions. Nat. Commun..

[96] Khattar E., Kumar P., Liu C.Y., Can Akincilar S., Raju A., Lakshmanan M., Maury J.J.P., Qiang Y., Li S., Tan E.Y. (2016). Telomerase Reverse Transcriptase Promotes Cancer Cell Proliferation by Augmenting TRNA Expression. J. Clin. Invest..

[97] Liu H., Liu Q., Ge Y., Zhao Q., Zheng X., Zhao Y. (2016). HTERT Promotes Cell Adhesion and Migration Independent of Telomerase Activity. Sci. Rep..

[98] Jaiswal R.K., Kumar P., Kumar M., Yadava P.K. (2018). HTERT Promotes Tumor Progression by Enhancing TSPAN13 Expression in Osteosarcoma Cells. Mol. Carcinog..

[99] Jaiswal R.K., Kumar P., Sharma A., Mishra D.K., Yadava P.K. (2017). Proteomic Identification of Proteins Differentially Expressed Following Overexpression of HTERT (Human Telomerase Reverse Transcriptase) in Cancer Cells. PLoS One.

[100] Maida Y., Yasukawa M., Furuuchi M., Lassmann T., Possemato R., Okamoto N., Kasim V., Hayashizaki Y., Hahn W.C., Masutomi K. (2009). An RNA-Dependent RNA Polymerase Formed by TERT and the RMRP RNA. Nature.

[101] Mattijssen S., Hinson E.R., Onnekink C., Hermanns P., Zabel B., Cresswell P., Pruijn G.J.M. (2011). Viperin MRNA Is a Novel Target for the Human RNase MRP/RNase P Endoribonuclease. Cell. Mol. Life Sci..

[102] Lemieux B., Laterreur N., Perederina A., Noël J.F., Dubois M.L., Krasilnikov A.S., Wellinger R.J. (2016). Active Yeast Telomerase Shares Subunits with Ribonucleoproteins RNase P and RNase MRP. Cell.

[103] Fujita T., Yuno M., Okuzaki D., Ohki R., Fujii H. (2015). Identification of Non-Coding RNAs Associated with Telomeres Using a Combination of EnChIP and RNA Sequencing. PLoS ONE.

[104] Santos J.H., Meyer J.N., Skorvaga M., Annab L.A., Van Houten B. (2004). Mitochondrial HTERT Exacerbates Free-Radical-Mediated MtDNA Damage. Aging Cell.

[105] Santos J.H., Meyer J.N., Van Houten B. (2006). Mitochondrial Localization of Telomerase as a Determinant for Hydrogen Peroxide-Induced Mitochondrial DNA Damage and Apoptosis. Hum. Mol. Genet..

[106] Saretzki G. (2014). Extra-Telomeric Functions of Human Telomerase: Cancer, Mitochondria and Oxidative Stress. Curr. Pharm. Des..

[107] Haendeler J., Dröse S., Büchner N., Jakob S., Altschmied J., Goy C., Spyridopoulos I., Zeiher A.M., Brandt U., Dimmeler S. (2009). Mitochondrial Telomerase Reverse Transcriptase Binds to and Protects Mitochondrial DNA and Function from Damage. Arterioscler. Thromb. Vasc. Biol..

[108] Naamati A., Regev-Rudzki N., Galperin S., Lill R., Pines O. (2009). Dual Targeting of Nfs1 and Discovery of Its Novel Processing Enzyme, Icp55. J. Biol. Chem..

[109] Haendeler J., Hoffmann J., Rahman S., Zeiher A.M., Dimmeler S. (2003). Regulation of Telomerase Activity and Anti-Apoptotic Function by Protein-Protein Interaction and Phosphorylation. FEBS Lett..

[110] Büchner N., Zschauer T.C., Lukosz M., Altschmied J., Haendeler J. (2010). Downregulation of Mitochondrial Telomerase Reverse Transcriptase Induced by H2O2 Is Src Kinase Dependent. Exp. Gerontol..

[111] Sharma N.K., Reyes A., Green P., Caron M.J., Bonini M.G., Gordon D.M., Holt I.J., Santos J.H. (2012). Human Telomerase Acts as a HTR-Independent Reverse Transcriptase in Mitochondria. Nucleic Acids Res..

[112] Chen L.Y., Zhang Y., Zhang Q., Li H., Luo Z., Fang H., Kim S.H., Qin L., Yotnda P., Xu J. (2012). Mitochondrial Localization of Telomeric Protein TIN2 Links Telomere Regulation to Metabolic Control. Mol. Cell.

[113] Kaguni L.S. (2004). DNA Polymerase Gamma, the Mitochondrial Replicase. Annu. Rev. Biochem..

[114] Balasubramaniam M., Reis R.J.S., Ayyadevara S., Wang X., Ganne A., Khaidakov M. (2017). Involvement of TRNAs in Replication of Human Mitochondrial DNA and Modifying Effects of Telomerase. Mech. Ageing Dev..

[115] Ahmed S., Passos J.F., Birket M.J., Beckmann T., Brings S., Peters H., Birch-Machin M.A., von Zglinicki T., Saretzki G. (2008). Telomerase Does Not Counteract Telomere Shortening but Protects Mitochondrial Function under Oxidative Stress. J. Cell Sci..

[116] Kondo S., Tanaka Y., Kondo Y., Hitomi M., Barnett G.H., Ishizaka Y., Liu J., Haqqi T., Nishiyama A., Villeponteau B. (1998). Antisense Telomerase Treatment: Induction of Two Distinct Pathways, Apoptosis and Differentiation. FASEB J..

[117] Folini M., Brambilla C., Villa R., Gandellini P., Vignati S., Paduano F., Daidone M.G., Zaffaroni N. (2005). Antisense Oligonucleotide-Mediated Inhibition of HTERT, but Not HTERC, Induces Rapid Cell Growth Decline and Apoptosis in the Absence of Telomere Shortening in Human Prostate Cancer Cells. Eur. J. Cancer.

[118] Massard C., Zermati Y., Pauleau A.L., Larochette N., Métivier D., Sabatier L., Kroemer G., Soria J.C. (2006). HTERT: A Novel Endogenous Inhibitor of the Mitochondrial Cell Death Pathway. Oncogene.

[119] Del Bufalo D., Rizzo A., Trisciuoglio D., Cardinali G., Torrisi M.R., Zangemeister-Wittke U., Zupi G., Biroccio A. (2005). Involvement of HTERT in Apoptosis Induced by Interference with Bcl-2 Expression and Function. Cell Death Differ..

[120] Gorbunova V., Seluanov A., Pereira-Smith O.M. (2003). Evidence That High Telomerase Activity May Induce a Senescent-like Growth Arrest in Human Fibroblasts. J. Biol. Chem..

[121] Kovalenko O.A., Caron M.J., Ulema P., Medrano C., Thomas A.P., Kimura M., Bonini M.G., Herbig U., Santos J.H. (2010). A Mutant Telomerase Defective in Nuclear-Cytoplasmic Shuttling Fails to Immortalize Cells and Is Associated with Mitochondrial Dysfunction. Aging Cell.

[122] Kovalenko O.A., Kaplunov J., Herbig U., detoledo S., Azzam E.I., Santos J.H. (2010). Expression of (NES-)HTERT in Cancer Cells Delays Cell Cycle Progression and Increases Sensitivity to Genotoxic Stress. PLoS One.

[123] Indran I.R., Hande M.P., Pervaiz S. (2011). HTERT Overexpression Alleviates Intracellular ROS Production, Improves Mitochondrial Function, and Inhibits ROS-Mediated Apoptosis in Cancer Cells. Cancer Res..

[124] Martens A., Schmid B., Akintola O., Saretzki G. (2019). Telomerase Does Not Improve DNA Repair in Mitochondria upon Stress but Increases MnSOD Protein under Serum-Free Conditions. Int. J. Mol. Sci..

[125] Zhang Z., Yu L., Dai G., Xia K., Liu G., Song Q., Tao C., Gao T., Guo W. (2017). Telomerase Reverse Transcriptase Promotes Chemoresistance by Suppressing Cisplatin-Dependent Apoptosis in Osteosarcoma Cells. Sci. Rep..

[126] Vonderheide R.H., Hahn W.C., Schultze J.L., Nadler L.M. (1999). The Telomerase Catalytic Subunit Is a Widely Expressed Tumor-Associated Antigen Recognized by Cytotoxic T Lymphocytes. Immunity.

[127] Hrdličková R., Nehyba J., Bose H.R. (2012). Alternatively Spliced Telomerase Reverse Transcriptase Variants Lacking Telomerase Activity Stimulate Cell Proliferation. Mol. Cell. Biol..

[128] Listerman I., Sun J., Gazzaniga F.S., Lukas J.L., Blackburn E.H. (2013). The Major Reverse Transcriptase-Incompetent Splice Variant of the Human Telomerase Protein Inhibits Telomerase Activity but Protects from Apoptosis. Cancer Res..

[129] Wick M., Zubov D., Hagen G. (1999). Genomic Organization and Promoter Characterization of the Gene Encoding the Human Telomerase Reverse Transcriptase (HTERT). Gene.

[130] Withers J.B., Ashvetiya T., Beemon K.L. (2012). Exclusion of Exon 2 Is a Common MRNA Splice Variant of Primate Telomerase Reverse Transcriptases. PLoS ONE.

[131] Zhu S., Rousseau P., Lauzon C., Gandin V., Topisirovic I., Autexier C. (2014). Inactive C-Terminal Telomerase Reverse Transcriptase Insertion Splicing Variants Are Dominant-Negative Inhibitors of Telomerase. Biochimie.

[132] Yi X., White D.M., Aisner D.L., Baur J.A., Wright W.E., Shay J.W. (2000). An Alternate Splicing Variant of the Human Telomerase Catalytic Subunit Inhibits Telomerase Activity. Neoplasia.

[133] Hisatomi H., Ohyashiki K., Ohyashiki J.H., Nagao K., Kanamaru T., Hirata H., Hibi N., Tsukada Y. (2003). Expression Profile of a Gamma-Deletion Variant of the Human Telomerase Reverse Transcriptase Gene. Neoplasia.

[134] Ulaner G.A., Hu J.-F., Vu T.H., Giudice L.C., Hoffman A.R. (1998). Telomerase Activity in Human Development Is Regulated by Human Telomerase Reverse Transcriptase (HTERT) Transcription and by Alternate Splicing of HTERT Transcripts. Cancer Res..

[135] Zhdanov D.D., Vasina D.A., Grachev V.A., Orlova E.V., Orlova V.S., Pokrovskaya M.V., Alexandrova S.S., Sokolov N.N. (2017). Alternative Splicing of Telomerase Catalytic Subunit HTERT Generated by Apoptotic Endonuclease EndoG Induces Human CD4 + T Cell Death. Eur. J. Cell Biol..

[136] Zhdanov D.D., Pokrovsky V.S., Orlova E.V., Orlova V.S., Pokrovskaya M.V., Aleksandrova S.S., Sokolov N.N. (2017). Intracellular Localization of Apoptotic Endonuclease EndoG and Splice-Variants of Telomerase Catalytic Subunit HTERT. Biochem. (Mosc.).

[137] Vařecha M., Potěšilová M., Matula P., Kozubek M. (2012). Endonuclease G Interacts with Histone H2B and DNA Topoisomerase II Alpha during Apoptosis. Mol. Cell. Biochem..

[138] Lee J.S., Seo T.W., Yi J.H., Shin K.S., Yoo S.J. (2013). CHIP Has a Protective Role against Oxidative Stress-Induced Cell Death through Specific Regulation of Endonuclease G. Cell Death Dis..

[139] Lee J.H., Khadka P., Baek S.H., Chung I.K. (2010). CHIP Promotes Human Telomerase Reverse Transcriptase Degradation and Negatively Regulates Telomerase Activity. J. Biol. Chem..

[140] Zhdanov D.D., Novachly N.S., Pokrovskaya M.V., Aleksandrova S.S., Kabardokov T.A., Sokolov N.N. (2020). Identification of Genes Whose MRNAs Are Subjected to Alternative Splicing by Endonuclease EndoG Action in Human and Murine CD4+ T Lymphocytes. Biomed. Chem. Res. Methods.

[141] Ludlow A.T., Wong M.S., Robin J.D., Batten K., Yuan L., Lai T.P., Dahlson N., Zhang L., Mender I., Tedone E. (2018). NOVA1 Regulates HTERT Splicing and Cell Growth in Non-Small Cell Lung Cancer. Nat. Commun..

[142] Li S., Crothers J., Haqq C.M., Blackburn E.H. (2005). Cellular and Gene Expression Responses Involved in the Rapid Growth Inhibition of Human Cancer Cells by RNA Interference-Mediated Depletion of Telomerase RNA. J. Biol. Chem..

[143] Ramakrishnan S.K., Varshney A., Sharma A., Das B.C., Yadava P.K. (2014). Expression of Targeted Ribozyme against Telomerase RNA Causes Altered Expression of Several Other Genes in Tumor Cells. Tumour Biol..

[144] Bagheri S., Nosrati M., Li S., Fong S., Torabian S., Rangel J., Moore D.H., Federman S., LaPosa R.R., Baehner F.L. (2006). Genes and Pathways Downstream of Telomerase in Melanoma Metastasis. Proc. Natl. Acad. Sci. USA.

[145] Liu H., Yang Y., Ge Y., Liu J., Zhao Y. (2019). TERC Promotes Cellular Inflammatory Response Independent of Telomerase. Nucleic Acids Res..

[146] Balakumaran A., Mishra P.J., Pawelczyk E., Yoshizawa S., Sworder B.J., Cherman N., Kuznetsov S.A., Bianco P., Giri N., Savage S.A. (2015). Bone Marrow Skeletal Stem/Progenitor Cell Defects in Dyskeratosis Congenita and Telomere Biology Disorders. Blood.

[147] Gazzaniga F.S., Blackburn E.H. (2014). An Antiapoptotic Role for Telomerase RNA in Human Immune Cells Independent of Telomere Integrity or Telomerase Enzymatic Activity. Blood.

[148] Sung L.Y., Chang W.F., Zhang Q., Liu C.C., Liou J.Y., Chang C.C., Ou-Yang H., Guo R., Fu H., Cheng W.T.K. (2014). Telomere Elongation and Naive Pluripotent Stem Cells Achieved from Telomerase Haplo-Insufficient Cells by Somatic Cell Nuclear Transfer. Cell Rep..

[149] Kedde M., Le Sage C., Duursma A., Zlotorynski E., Van Leeuwen B., Nijkamp W., Beijersbergen R., Agami R. (2006). Telomerase-Independent Regulation of ATR by Human Telomerase RNA. J. Biol. Chem..

[150] Chu C., Qu K., Zhong F.L., Artandi S.E., Chang H.Y. (2011). Genomic Maps of Long Noncoding RNA Occupancy Reveal Principles of RNA-Chromatin Interactions. Mol. Cell.

[151] Ivanyi-Nagy R., Ahmed S.M., Peter S., Ramani P.D., Ong P.F., Dreesen O., Dröge P. (2018). The RNA Interactome of Human Telomerase RNA Reveals a Coding-Independent Role for a Histone MRNA in Telomere Homeostasis. Elife.

[152] Trapp S., Parcells M.S., Kamil J.P., Schumacher D., Tischer B.K., Kumar P.M., Nair V.K., Osterrieder N. (2006). A Virus-Encoded Telomerase RNA Promotes Malignant T Cell Lymphomagenesis. J. Exp. Med..

[153] Kaufer B.B., Arndt S., Trapp S., Osterrieder N., Jarosinski K.W. (2011). Herpesvirus Telomerase RNA (VTR) with a Mutated Template Sequence Abrogates Herpesvirus-Induced Lymphomagenesis. PLoS Pathog..

[154] Kheimar A., Trimpert J., Groenke N., Kaufer B.B. (2019). Overexpression of Cellular Telomerase RNA Enhances Virus-Induced Cancer Formation. Oncogene.

[155] Kaufer B.B., Trapp S., Jarosinski K.W., Osterrieder N. (2010). Herpesvirus Telomerase RNA(VTR)-Dependent Lymphoma Formation Does Not Require Interaction of VTR with Telomerase Reverse Transcriptase (TERT). PLoS Pathog..

[156] Le S., Sternglanz R., Greider C.W. (2000). Identification of Two RNA-Binding Proteins Associated with Human Telomerase RNA. Mol. Biol. Cell.

[157] Kheimar A., Kaufer B.B. (2018). Epstein-Barr Virus-Encoded RNAs (EBERs) Complement the Loss of Herpesvirus Telomerase RNA (VTR) in Virus-Induced Tumor Formation. Sci. Rep..

[158] Tycowski K.T., Guo Y.E., Lee N., Moss W.N., Vallery T.K., Xie M., Steitz J.A. (2015). Viral Noncoding RNAs: More Surprises. Genes Dev..

[159] Lees-Miller S.P., Meek K. (2003). Repair of DNA Double Strand Breaks by Non-Homologous End Joining. Biochimie.

[160] Stellwagen A.E., Haimberger Z.W., Veatch J.R., Gottschling D.E. (2003). Ku Interacts with Telomerase RNA to Promote Telomere Addition at Native and Broken Chromosome Ends. Genes Dev..

[161] Ting N.S.Y., Yu Y., Pohorelic B., Lees-Miller S.P., Beattie T.L. (2005). Human Ku70/80 Interacts Directly with HTR, the RNA Component of Human Telomerase. Nucleic Acids Res..

[162] Pfingsten J.S., Goodrich K.J., Taabazuing C., Ouenzar F., Chartrand P., Cech T.R. (2012). Mutually Exclusive Binding of Telomerase RNA and DNA by Ku Alters Telomerase Recruitment Model. Cell.

[163] Anisenko A.N., Knyazhanskaya E.S., Zatsepin T.S., Gottikh M.B. (2017). Human Ku70 Protein Binds Hairpin RNA and Double Stranded DNA through Two Different Sites. Biochimie.

[164] Chai W., Ford L.P., Lenertz L., Wright W.E., Shay J.W. (2002). Human Ku70/80 Associates Physically with Telomerase through Interaction with HTERT. J. Biol. Chem..

[165] Tomlinson R.L., Abreu E.B., Ziegler T., Ly H., Counter C.M., Terns R.M., Terns M.P. (2008). Telomerase Reverse Transcriptase Is Required for the Localization of Telomerase RNA to Cajal Bodies and Telomeres in Human Cancer Cells. Mol. Biol. Cell.

[166] Fiset S., Chabot B. (2001). HnRNP A1 May Interact Simultaneously with Telomeric DNA and the Human Telomerase RNA in Vitro. Nucleic Acids Res..

[167] Ting N.S.Y., Pohorelic B., Yu Y., Lees-Miller S.P., Beattie T.L. (2009). The Human Telomerase RNA Component, HTR, Activates the DNA-Dependent Protein Kinase to Phosphorylate Heterogeneous Nuclear Ribonucleoprotein A1. Nucleic Acids Res..

[168] Sui J., Lin Y.F., Xu K., Lee K.J., Wang D., Chen B.P.C. (2015). DNA-PKcs Phosphorylates HnRNP-A1 to Facilitate the RPA-to-POT1 Switch and Telomere Capping after Replication. Nucleic Acids Res..

[169] Raghunandan M., Geelen D., Majerova E., Decottignies A. (2021). NHP2 Downregulation Counteracts HTR-Mediated Activation of the DNA Damage Response at ALT Telomeres. EMBO J..

[170] Flynn R.L., Cox K.E., Jeitany M., Wakimoto H., Bryll A.R., Ganem N.J., Bersani F., Pineda J.R., Suvà M.L., Benes C.H. (2015). Alternative Lengthening of Telomeres Renders Cancer Cells Hypersensitive to ATR Inhibitors. Science.

[171] Vulliamy T., Beswick R., Kirwan M., Marrone A., Digweed M., Walne A., Dokal I. (2008). Mutations in the Telomerase Component NHP2 Cause the Premature Ageing Syndrome Dyskeratosis Congenita. Proc. Natl. Acad. Sci. USA.

[172] Cesare A.J., Kaul Z., Cohen S.B., Napier C.E., Pickett H.A., Neumann A.A., Reddel R.R. (2009). Spontaneous Occurrence of Telomeric DNA Damage Response in the Absence of Chromosome Fusions. Nat. Struct. Mol. Biol..

[173] Amato R., Valenzuela M., Berardinelli F., Salvati E., Maresca C., Leone S., Antoccia A., Sgura A. (2020). G-Quadruplex Stabilization Fuels the ALT Pathway in ALT-Positive Osteosarcoma Cells. Genes.

[174] Chang D.D., Clayton D.A. (1989). Mouse RNAase MRP RNA Is Encoded by a Nuclear Gene and Contains a Decamer Sequence Complementary to a Conserved Region of Mitochondrial RNA Substrate. Cell.

[175] Alfonzo J.D., Söll D. (2009). Mitochondrial TRNA Import--the Challenge to Understand Has Just Begun. Biol. Chem..

[176] Wang G., Chen H.W., Oktay Y., Zhang J., Allen E.L., Smith G.M., Fan K.C., Hong J.S., French S.W., McCaffery J.M. (2010). PNPASE Regulates RNA Import into Mitochondria. Cell.

[177] Mercer T.R., Neph S., Dinger M.E., Crawford J., Smith M.A., Shearwood A.M.J., Haugen E., Bracken C.P., Rackham O., Stamatoyannopoulos J.A. (2011). The Human Mitochondrial Transcriptome. Cell.

[178] Zhang X., Zuo X., Yang B., Li Z., Xue Y., Zhou Y., Huang J., Zhao X., Zhou J., Yan Y. (2014). MicroRNA Directly Enhances Mitochondrial Translation during Muscle Differentiation. Cell.

[179] Wang G., Shimada E., Koehler C.M., Teitell M.A. (2012). PNPASE and RNA Trafficking into Mitochondria. Biochim. Biophys. Acta.

[180] Cheng Y., Liu P., Zheng Q., Gao G., Yuan J., Wang P., Huang J., Xie L., Lu X., Tong T. (2018). Mitochondrial Trafficking and Processing of Telomerase RNA TERC. Cell Rep..

[181] Denesyuk N.A., Thirumalai D. (2011). Crowding Promotes the Switch from Hairpin to Pseudoknot Conformation in Human Telomerase RNA. J. Am. Chem. Soc..

[182] Liu P., Huang J., Zheng Q., Xie L., Lu X., Jin J., Wang G. (2017). Mammalian Mitochondrial RNAs Are Degraded in the Mitochondrial Intermembrane Space by RNASET2. Protein Cell.

[183] Luhtala N., Parker R. (2010). T2 Family Ribonucleases: Ancient Enzymes with Diverse Roles. Trends Biochem. Sci..

[184] Hillwig M.S., Contento A.L., Meyer A., Ebany D., Bassham D.C., MacIntosha G.C. (2011). RNS2, a Conserved Member of the RNase T2 Family, Is Necessary for Ribosomal RNA Decay in Plants. Proc. Natl. Acad. Sci. USA.

[185] Zheng Q., Liu P., Gao G., Yuan J., Wang P., Huang J., Xie L., Lu X., Di F., Tong T. (2019). Mitochondrion-Processed TERC Regulates Senescence without Affecting Telomerase Activities. Protein Cell.

[186] Nicholls C., Pinto A.R., Li H., Li N., Wang L., Simpson R., Liu J.P. (2012). Glyceraldehyde-3-Phosphate Dehydrogenase (GAPDH) Induces Cancer Cell Senescence by Interacting with Telomerase RNA Component. Proc. Natl. Acad. Sci. USA.

[187] Sawa A., Khan A.A., Hester L.D., Snyder S.H. (1997). Glyceraldehyde-3-Phosphate Dehydrogenase: Nuclear Translocation Participates in Neuronal and Nonneuronal Cell Death. Proc. Natl. Acad. Sci. USA.

[188] Hara M.R., Agrawal N., Kim S.F., Cascio M.B., Fujimuro M., Ozeki Y., Takahashi M., Cheah J.H., Tankou S.K., Hester L.D. (2005). S-Nitrosylated GAPDH Initiates Apoptotic Cell Death by Nuclear Translocation Following Siah1 Binding. Nat. Cell Biol..

[189] Sen N., Hara M.R., Kornberg M.D., Cascio M.B., Bae B.I., Shahani N., Thomas B., Dawson T.M., Dawson V.L., Snyder S.H. (2008). Nitric Oxide-Induced Nuclear GAPDH Activates P300/CBP and Mediates Apoptosis. Nat. Cell Biol..

[190] Chuang D.M., Ishitani R., Roses A.D., Burke J.R., Vance J.M., Strittmatter W.J. (1996). A Role for GAPDH in Apoptosis and Neurodegeneration. Nat. Med..

[191] Nagy E., Henics T., Eckert M., Miseta A., Lightowlers R.N., Kellermayer M. (2000). Identification of the NAD(+)-Binding Fold of Glyceraldehyde-3-Phosphate Dehydrogenase as a Novel RNA-Binding Domain. Biochem. Biophys. Res. Commun..

[192] Azam S., Jouvet N., Jilani A., Vongsamphanh R., Yang X., Yang S., Ramotar D. (2008). Human Glyceraldehyde-3-Phosphate Dehydrogenase Plays a Direct Role in Reactivating Oxidized Forms of the DNA Repair Enzyme APE1. J. Biol. Chem..

[193] Chang C., Su H., Zhang D., Wang Y., Shen Q., Liu B., Huang R., Zhou T., Peng C., Wong C.C.L. (2015). AMPK-Dependent Phosphorylation of GAPDH Triggers Sirt1 Activation and Is Necessary for Autophagy upon Glucose Starvation. Mol. Cell.

[194] Fish L., Zhang S., Yu J.X., Culbertson B., Zhou A.Y., Goga A., Goodarzi H. (2018). Cancer Cells Exploit an Orphan RNA to Drive Metastatic Progression. Nat. Med..

[195] Laudadio I., Orso F., Azzalin G., Calabrò C., Berardinelli F., Coluzzi E., Gioiosa S., Taverna D., Sgura A., Carissimi C. (2019). AGO2 Promotes Telomerase Activity and Interaction between the Telomerase Components TERT and TERC. EMBO Rep..

[196] Laudadio I., Carissimi C., Fulci V. (2019). How RNAi Machinery Enters the World of Telomerase. Cell Cycle.

[197] Rubtsova M., Naraykina Y., Vasilkova D., Meerson M., Zvereva M., Prassolov V., Lazarev V., Manuvera V., Kovalchuk S., Anikanov N. (2018). Protein Encoded in Human Telomerase RNA Is Involved in Cell Protective Pathways. Nucleic Acids Res..

[198] Hartford C.C.R., Lal A. (2020). When Long Noncoding Becomes Protein Coding. Mol. Cell. Biol..

[199] Tseng C.K., Wang H.F., Burns A.M.M., Schroeder M.R.R., Gaspari M., Baumann P. (2015). Human Telomerase RNA Processing and Quality Control. Cell Rep..

[200] Mudge J.M., Jungreis I., Hunt T., Gonzalez J.M., Wright J.C., Kay M., Davidson C., Fitzgerald S., Seal R., Tweedie S. (2019). Discovery of High-Confidence Human Protein-Coding Genes and Exons by Whole-Genome PhyloCSF Helps Elucidate 118 GWAS Loci. Genome Res..

[201] van Heesch S., Witte F., Schneider-Lunitz V., Schulz J.F., Adami E., Faber A.B., Kirchner M., Maatz H., Blachut S., Sandmann C.L. (2019). The Translational Landscape of the Human Heart. Cell.

[202] Ruiz-Orera J., Albà M.M. (2019). Conserved Regions in Long Non-Coding RNAs Contain Abundant Translation and Protein-RNA Interaction Signatures. NAR genomics Bioinforma..

[203] Nguyen D., Grenier St-Sauveur V., Bergeron D., Dupuis-Sandoval F., Scott M.S.S., Bachand F. (2015). A Polyadenylation-Dependent 3’ End Maturation Pathway Is Required for the Synthesis of the Human Telomerase RNA. Cell Rep..

[204] Ingolia N.T., Brar G.A., Stern-Ginossar N., Harris M.S., Talhouarne G.J.S., Jackson S.E., Wills M.R., Weissman J.S. (2014). Ribosome Profiling Reveals Pervasive Translation Outside of Annotated Protein-Coding Genes. Cell Rep..

[205] Brenner K.A. (2019). A Noncanonical Function of the Telomerase RNA Component in Human Embryonic Stem Cells. Arts Sci. Electron. Theses Diss..

[206] Feng J., Funk W.D., Wang S.S., Weinrich S.L., Avilion A.A., Chiu C.P., Adams R.R., Chang E., Allsopp R.C., Yu J. (1995). The RNA Component of Human Telomerase. Science.

[207] Hoare S.F., Bryce L.A., Bea G., Wisman A., Burns S., Going J.J., Van Der Zee A.G.J., Keith W.N. (2001). Lack of Telomerase RNA Gene HTERC Expression in Alternative Lengthening of Telomeres Cells Is Associated with Methylation of the HTERC Promoter 1. CANCER Res..

[208] Henson J.D., Neumann A.A., Yeager T.R., Reddel R.R. (2002). Alternative Lengthening of Telomeres in Mammalian Cells. Oncogene.

[209] Herbig U., Jobling W.A., Chen B.P.C., Chen D.J., Sedivy J.M. (2004). Telomere Shortening Triggers Senescence of Human Cells through a Pathway Involving ATM, P53, and P21(CIP1), but Not P16(INK4a). Mol. Cell.

[210] Zimmermann S., Voss M., Kaiser S., Kapp U., Waller C.F., Martens U.M. (2003). Lack of Telomerase Activity in Human Mesenchymal Stem Cells. Leukemia.

[211] Baena-Del Valle J.A., Zheng Q., Esopi D.M., Rubenstein M., Hubbard G.K., Moncaliano M.C., Hruszkewycz A., Vaghasia A., Yegnasubramanian S., Wheelan S.J. (2018). MYC Drives Overexpression of Telomerase RNA (HTR/TERC) in Prostate Cancer. J. Pathol..

[212] Kang S.S., Kwon T., Kwon D.Y., Do S. (1999). Il Akt Protein Kinase Enhances Human Telomerase Activity through Phosphorylation of Telomerase Reverse Transcriptase Subunit. J. Biol. Chem..

[213] Wu S., Ge Y., Lin K., Liu Q., Zhou H., Hu Q., Zhao Y., He W., Ju Z. (2022). Telomerase RNA TERC and the PI3K-AKT Pathway Form a Positive Feedback Loop to Regulate Cell Proliferation Independent of Telomerase Activity. Nucleic Acids Res..

[214] Cerezo A., Kalthoff H., Schuermann M., Schäfer B., Boukamp P. (2002). Dual Regulation of Telomerase Activity through C-Myc-Dependent Inhibition and Alternative Splicing of HTERT. J. Cell Sci..

[215] Jo D., Park R., Kim H., Jang M., Lee E.J., Jang I.S., Park J. (2018). AMP-Activated Protein Kinase Regulates the Expression of Human Telomerase Reverse Transcriptase. PLoS One.

[216] Karnewar S., Neeli P.K., Panuganti D., Kotagiri S., Mallappa S., Jain N., Jerald M.K., Kotamraju S. (2018). Metformin Regulates Mitochondrial Biogenesis and Senescence through AMPK Mediated H3K79 Methylation: Relevance in Age-Associated Vascular Dysfunction. Biochim. Biophys. Acta. Mol. Basis Dis..

[217] McKinney A., Amen A., Stevers N., Costello J. (2019). CSIG-24. Gabp Links Ampk Signaling to Tert Regulation in a Tert Promoter Mutation Dependent Manner. Neuro. Oncol..

[218] Kwon H.J., Rhim J.H., Jang I.S., Kim G.E., Park S.C., Yeo E.J. (2010). Activation of AMP-Activated Protein Kinase Stimulates the Nuclear Localization of Glyceraldehyde 3-Phosphate Dehydrogenase in Human Diploid Fibroblasts. Exp. Mol. Med..

[219] Sundararaj K.P., Wood R.E., Ponnusamy S., Salas A.M., Szulc Z., Bielawska A., Obeid L.M., Hannun Y.A., Ogretmen B. (2004). Rapid Shortening of Telomere Length in Response to Ceramide Involves the Inhibition of Telomere Binding Activity of Nuclear Glyceraldehyde-3-Phosphate Dehydrogenase. J. Biol. Chem..

[220] Demarse N.A., Ponnusamy S., Spicer E.K., Apohan E., Baatz J.E., Ogretmen B., Davies C. (2009). Direct Binding of Glyceraldehyde 3-Phosphate Dehydrogenase to Telomeric DNA Protects Telomeres against Chemotherapy-Induced Rapid Degradation. J. Mol. Biol..

[221] Pariona-Llanos R., Pavani R.S., Reis M., Noël V., Silber A.M., Armelin H.A., Cano M.I.N., Elias M.C. (2015). Glyceraldehyde 3-Phosphate Dehydrogenase-Telomere Association Correlates with Redox Status in Trypanosoma Cruzi. PLoS One.

[222] Abdel-Haleem A.M., Lewis N.E., Jamshidi N., Mineta K., Gao X., Gojobori T. (2017). The Emerging Facets of Non-Cancerous Warburg Effect. Front. Endocrinol..

[223] Cho Y.M., Kwon S., Pak Y.K., Seol H.W., Choi Y.M., Park D.J., Park K.S., Lee H.K. (2006). Dynamic Changes in Mitochondrial Biogenesis and Antioxidant Enzymes during the Spontaneous Differentiation of Human Embryonic Stem Cells. Biochem. Biophys. Res. Commun..

[224] Folmes C.D.L., Nelson T.J., Martinez-Fernandez A., Arrell D.K., Lindor J.Z., Dzeja P.P., Ikeda Y., Perez-Terzic C., Terzic A. (2011). Somatic Oxidative Bioenergetics Transitions into Pluripotency-Dependent Glycolysis to Facilitate Nuclear Reprogramming. Cell Metab..

[225] Rodic S., Vincent M.D. (2018). Reactive Oxygen Species (ROS) Are a Key Determinant of Cancer’s Metabolic Phenotype. Int. J. cancer.

[226] Ren Y., Shen H.M. (2019). Critical Role of AMPK in Redox Regulation under Glucose Starvation. Redox Biol..

[227] Fujita T., Asano Y., Ohtsuka J., Takada Y., Saito K., Ohki R., Fujii H. (2013). Identification of Telomere-Associated Molecules by Engineered DNA-Binding Molecule-Mediated Chromatin Immunoprecipitation (EnChIP). Sci. Rep..

[228] Déjardin J., Kingston R.E. (2009). Purification of Proteins Associated with Specific Genomic Loci. Cell.

[229] Goldfarb K.C. (2016). Noncoding MRP RNA Function Investigated by Genetic Manipulation and Biochemical Analysis. Ph.D. Thesis.

[230] Wu Y.L., Dudognon C., Nguyen E., Hillion J., Pendino F., Tarkanyi I., Aradi J., Lanotte M., Tong J.H., Chen G.Q. (2006). Immunodetection of Human Telomerase Reverse-Transcriptase (HTERT) Re-Appraised: Nucleolin and Telomerase Cross Paths. J. Cell Sci..

