# Supplementary material for: Many Functions of Telomerase Components: Certainties, Doubts, and Inconsistencies
Source: Int J Mol Sci. 2022 Dec 2;23(23):15189. doi: 10.3390/ijms232315189 (PMC9736166; doi:10.3390/ijms232315189)
Supplement: Supplementary file 1 [file ijms-23-15189-s001.zip › ijms-2021861-supplementary.pdf]

NON-TELOMERIC FUNCTIONS OF THE TELOMERASE COMPONENTS TERT AND TERC: CERTAINTIES, DOUBTS, AND INCONSISTENCIES

Ion Udroiu, Jessica Marinaccio, Antonella Sgura

SUPPLEMENTARY MATERIAL

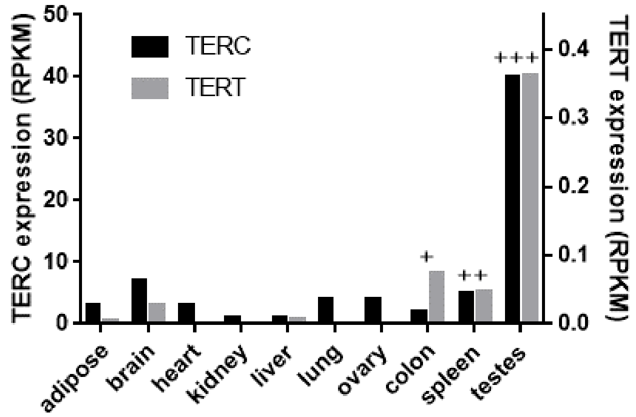

Figure S1 – Expression of TERC and TERT in human adult tissues. Data taken from Castle et al. (2010) and Fagerberg et al. (2014). Telomerase activity is indicated as high (+++), medium (++), low (+) or absent (no sign).

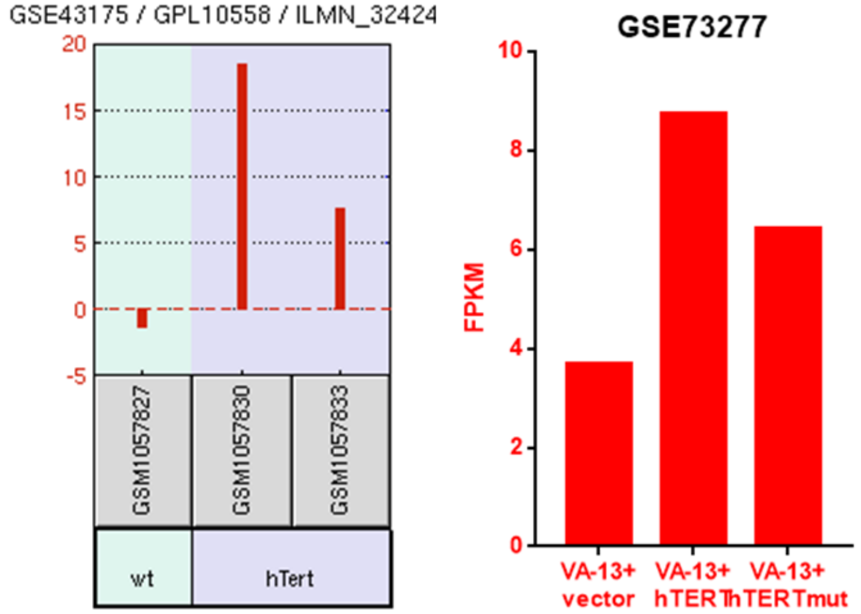

Figure S2 – Expression of RMRP in normal and hTert-expressing fibroblasts. Graph on the left shows differences between wt (green) and hTert-expressing IMR90 fibroblasts and is generated by the GEO2R interactive web tool (www.ncbi.nlm.nih.gov/geo/geo2r/) using dataset GSE43175; data are log-transformed. Graph on the right shows differences between wt and hTert-expressing VA13 fibroblasts; data taken from dataset GSE43175.

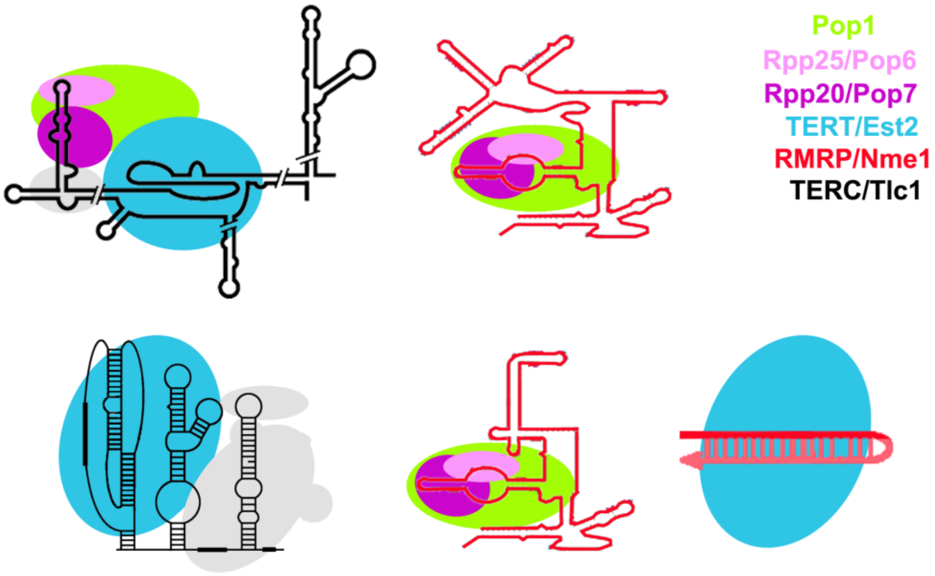

Figure S3 – Links between telomerase and RNase MRP. In yeast, Pop1, Pop6 and Pop7 are part both of the telomerase (a) and the RNAse MRP (b) complex. Pop6 and Pop7 recognize a stem which is similar in the RNA component of telomerase (Tlc1, ortholog of TERC) and in the RNA component of RNAse MRP (Nme1, ortholog of RMRP). In vertebrates, TERC does not bind to Pop1, Rpp25 (ortholog of Pop6) and Rpp20 (ortholog of Pop7). Maida et al. (2009) proposed that human TERT act as an RNA-dependent RNA polymerase, elongating RMRP to form a dsRNA (e).

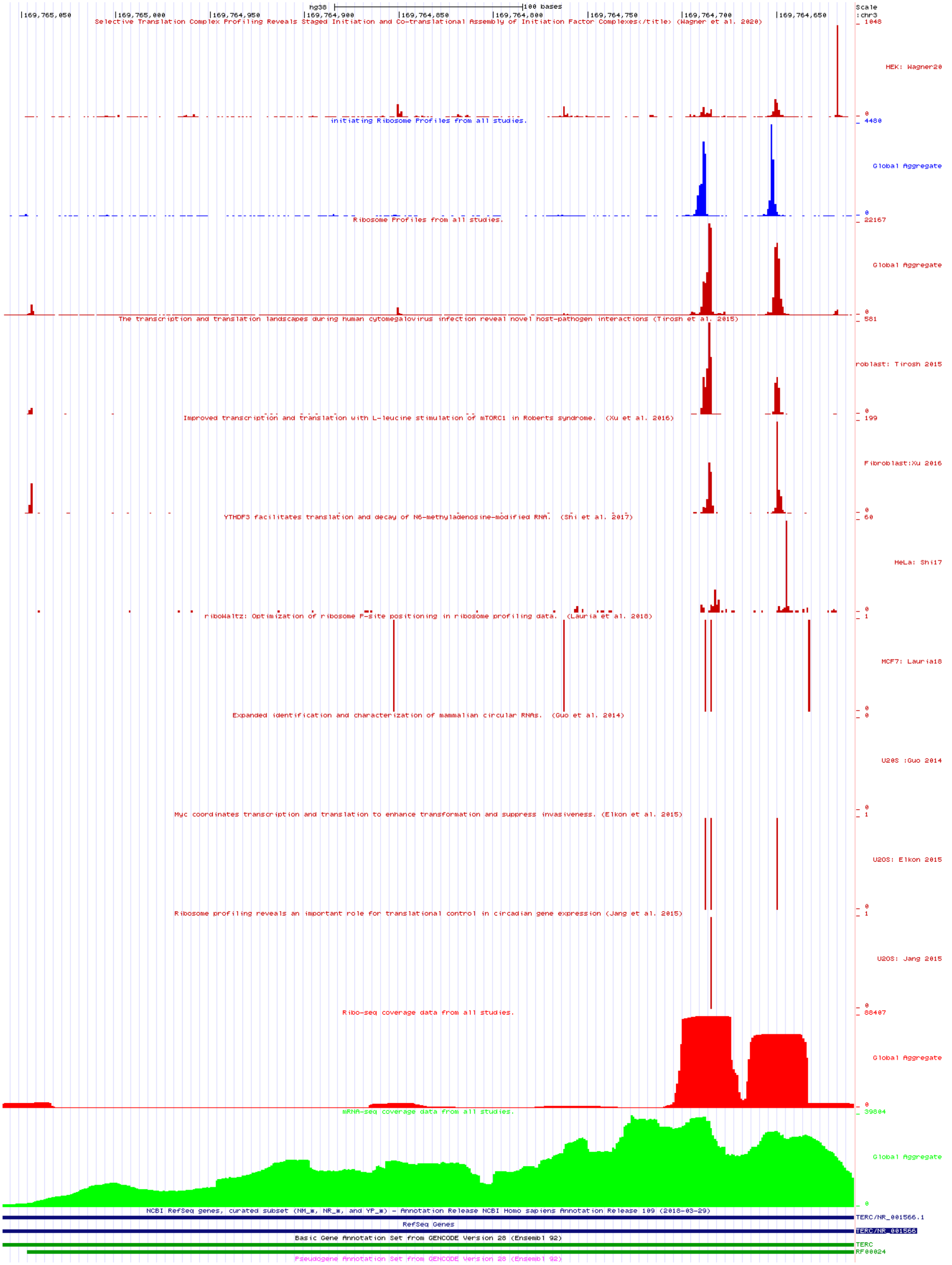

Figure S4 – Ribosome profiling of TERC. The image can be accessed on the GWIPs-viz browser (https://gwips.ucc.ie/cgi-bin/hgTracks?db=hg38&lastVirtModeType=default&lastVirtModeExtraState=&virtModeType=default&virtMode=0&nonVirtPosition=&position=chr3%3A169764610%2D169765060&hgsid=122433_X4ewa0PG4Q4QHFBAUt9El5YaBqFv).

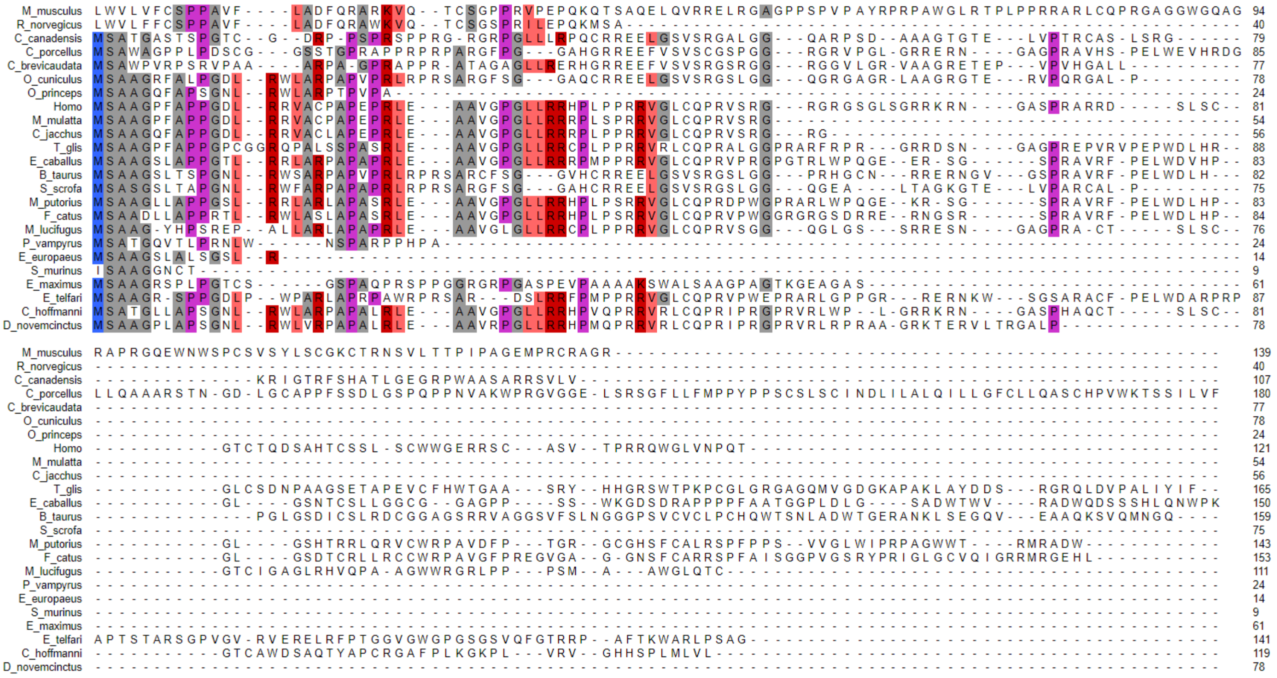

Figure S5 – Alignment of putative TERP orthologues.

Upregulated by TERT	Downregulated by TERT
Adhesion and migration pathway[1]	Differentiation pathway (including keratins)[2]
Wnt- and TGF-b pathways[2]	
CDKN2B[1,2]	
Hsp60, *Hsp70*, *Hsp90*[3]	
GAPDH[3]	
	NEAT1, *MALAT1* (lncRNAs) [1]

Table S1 – Pathways and genes regulated by TERT in TERC-negative cells. Data from: 1: Liu et al. (2016); 2: Jaiswal et al. (2018); 3: Jaiwal et al. (2017).

Upregulated by TERC	Downregulated by TERC
Glycolytic pathway[1,2]	*TIGAR* (inhibitor of glycolysis)[4]
Cellular immunity pathway[3]	
Cyclins[2,6], CDKs[2,3], Aurora Kinases[2], *HAUS1*[4,5]	p53/apoptosis pathways[2,4]
Oncogenes (*MYB*[2], *MET*[6])	Oncogene (*KRAS*[2])
DNA replication pathway[2,7]	
DNA repair (*BRCA2*[1,4], *RECQL*[4], *WRN*[2])	DNA damage checkpoints (*ATM*[2,7], *ATR*[4], *ATRIP*[5])
Chromatin regulators (*HDAC2*[1], *HDAC3*[2], *HDAC7*[2], *KMT5A*[2], *DOT1L*[4], *KDM5A*[4], *ATRX*[4], *DAXX*[4])	
Transcription factors (*HMGA1*[1,4,5], *GABPA*[4], *GABPB1*[2])	
Ribosome 60S genes (including *RPL22*)[2,5]	
RNASET2[4]	
	Keratins[2,3,5]

Table S2 – Pathways and genes regulated by TERC. Data from: 1: Bagheri et al. (2006); 2: Gazzaniga and Blackburn (2014); 3: Liu et al. (2019); 4: Balakumaran et al. (2015); 5: Ramakrishnan et al. (2014); 6: Li et al. (2005); 7: Sung et al. (2014); 8: Kedde et al. (2006).
